# Locus- and Gene-Level Insights into the Inverse Association Between Alzheimer’s Disease and Cancer

**DOI:** 10.3390/ijms27062900

**Published:** 2026-03-23

**Authors:** Dipti Debnath, Mohammad Housini, Sanjeev Sariya, Nicole R. Phillips, Gita A. Pathak, Robert C. Barber

**Affiliations:** 1 Department of Microbiology, Immunology & Genetics, University of North Texas Health Science Center, 3500 Camp Bowie Blvd, Fort Worth, TX 76107, USA; 2 Institute for Translational Research, University of North Texas Health Science Center, 3500 Camp Bowie Blvd, Fort Worth, TX 76107, USA; 3 Institute for Genomic Health, Icahn School of Medicine at Mount Sinai, 1 Gustave L. Levy Pl, New York, NY 10029, USA; 4 Department of Genetics and Genomic Sciences, Icahn School of Medicine at Mount Sinai, 1 Gustave L. Levy Pl, New York, NY 10029, USA

**Keywords:** apoptosis, APOE, local genetic correlation, genetically regulated gene expression, inverse comorbidity

## Abstract

Alzheimer’s disease (AD) and cancer are both age-related conditions, yet numerous large-scale epidemiological studies have consistently documented an inverse association, with individuals diagnosed with cancer exhibiting a reduced risk of AD and vice versa. Although this relationship has been replicated across diverse populations, its biological basis remains poorly understood. To address this gap, the present study applies a framework that integrates locus-level genetic correlation (rg) with genetically regulated gene expression to clarify the molecular factors contributing to the inverse epidemiological patterns observed between the two diseases. We used the largest available genome-wide association studies (GWAS) (Nmax = 448,150) to quantify local genetic correlations between AD and several age-associated cancers, including breast, prostate, lung, colorectal, melanoma, basal cell carcinoma, bladder, and endometrial cancer. Eight genomic regions showed significant negative local rg, at the 19q13.31–19q13.32 locus demonstrating strong negative correlations across multiple cancers, including breast, prostate, lung, melanoma, and endometrial cancer. To evaluate the contribution of genetically regulated gene expression, we conducted transcriptome-wide association studies (TWAS) using precomputed gene expression weights from cancer tissues (The Cancer Genome Atlas-TCGA), disease-agnostic tissues (Genotype-Tissue Expression-GTEx), and brain tissue (dorsolateral prefrontal cortex-DLPFC). For each AD–cancer pair, we prioritized genes that were nominally significant in both traits (*p* < 0.05) and exhibited inverse TWAS Z scores. This analysis identified 24 genes with opposite effect directions between AD and at least three cancer types. TWAS signals also aligned with local rg findings at the 19q13.31–19q13.32 region, suggesting that regulatory variation near this locus contributes to shared but opposing genetic effects beyond the canonical APOE signal. Across cancer types, genes inversely associated with AD converged on pathways involved in cell cycle regulation, apoptosis, DNA-damage response, and metabolic processes. These results support the hypothesis that biological mechanisms promoting proliferation and survival in cancer may oppose those contributing to neurodegeneration in AD.

## 1. Introduction

Alzheimer’s disease (AD) and cancer are among the leading causes of mortality and morbidity worldwide. AD is the predominant form of dementia and is a progressively advancing chronic neurodegenerative condition, mostly impacting elderly persons and resulting in gradual deterioration of cognitive function, impaired memory, and alteration in behavior [1]. The predominant neuropathological features of AD include senile plaques, which are composed of extracellular accumulations of amyloid beta (Aβ) plaque, and neurofibrillary tangles, which are intracellular accumulations of an abnormally phosphorylated microtubule-associated protein tau (MAPT) [1,2]. AD is the 5th most common cause of death in the US, accounting for 119,399 recorded deaths in 2021 and an expected 6.9 million frequent cases among those 65 and older in 2024 [3]. Cancer is the second-most common cause of death altogether and the most significant incidence for those under the age of 85 in the US [4]. In 2025, projections indicate 618,120 cancer deaths and 2,041,910 new cancer cases in the United States [5]. Cancer refers to a collection of malignancies distinguished by unregulated proliferation and division of cells. In contrast to AD, which is characterized by neuronal death, cancer is propelled by mutations that allow cells to avoid apoptosis, leading to their malignant expansion. The generation of genetic and epigenetic changes that dysregulate critical functions in cells propels the progression of cancer. Uncontrolled proliferation results from mutations in oncogenes (like *KRAS* and *MYC*), while cell cycle regulation and apoptosis are compromised by the loss of tumor suppressor genes (like *TP53*, *RB1*, and *PTEN*). Furthermore, uncontrolled growth, invasion, and metastasis are caused by disruption of intercellular communication via modified signaling pathways, including growth factor, cytokine, and cell adhesion networks [6,7].

AD and cancer are both linked to aging, but certain epidemiological studies have reported a negative association between the two diseases [8,9,10]. Studies report that those with cancer are less prone to get AD, and vice versa. Driver and colleagues (2012) reported that tumor patients had a 35% lower likelihood of being diagnosed with AD. On the other side, the probability of cancer development was 43% lower in AD patients [11]. In addition, Musicco and colleagues (2013) noted in their population-based (N > 1,000,000) research that people with AD had a 50% lower risk of cancer and a 35% reduced risk of AD in cancer patients [12]. Although it has been reliably replicated, the etiological basis of this negative association between cancer and AD remains unclear. This may be attributed to shared biological mechanisms between the two diseases or because of the pharmaceutical treatments that the patients in the studies received. Many shared genes and pathways showed increased expression in AD but were inhibited in prostate, lung, and colorectal cancers and vice versa, according to a transcriptomic meta-analysis that included gene expression results from related tissues [13]. In addition, it has been proven that cancer and AD have unique patterns of microRNA expression [14]. Cancer cells and degenerating neurons have been shown to control several similar proteins (such as Pin and P53) and pathways (e.g., Wnt cell survival pathway) in distinctive approaches [15,16,17].

Genome-wide association studies (GWAS) have provided details on single nucleotide polymorphisms (SNPs) linked to AD and cancer [18,19]. Several genetic studies have been conducted to explain the inverse association between cancer and AD. Feng and colleagues (2017) [20] investigated genetic correlations between AD and five cancer types (colon, breast, prostate, ovarian, and lung), reporting positive genetic associations between AD and lung and breast, and insignificant results for other cancers. They also reported inverse associations at enhancer and repressor sites, suggesting variants involved in shared regulatory systems with opposing genetic effects. Seddighi and colleagues (2019) [21] used a two-sample Mendelian randomization (MR) approach and observed that genetically predicted risks for breast, leukemia, and lung cancers significantly lower the risk of AD. In general, a greater genetic susceptibility to cancer, particularly malignancies connected to smoking, was causally associated with a lower chance of getting AD. In another study, Dong and colleagues (2023) [22] used an MR approach to report that genetic susceptibility to AD decreases cancer risk (N = 651,985) mediated by very low-density lipoprotein (VLDL). While *PVRIG* overexpression improves antitumor immunity and cancer survival, it also raises the risk of AD. Using transcriptome data from 33 TCGA cancer types, they also conducted colocalization analysis to identify genes whose elevated expression is a risk factor for AD. In a two-stage experiment using 9576 participants, Pathak and colleagues (2020) [23] compared sex-stratified participants diagnosed with cancer or AD to controls, adopting Bayesian multinomial regression [23] to identify SNPs inversely linked to AD and two age-related malignancies (breast and prostate cancer). On chromosomes 4, 5 and 19 they discovered 137 variants with inverse odds ratios for cancer and AD. The miRNAs mapped inside the network were shown to be enriched for the miR-17 and miR-515 families.

**Figure 1:**
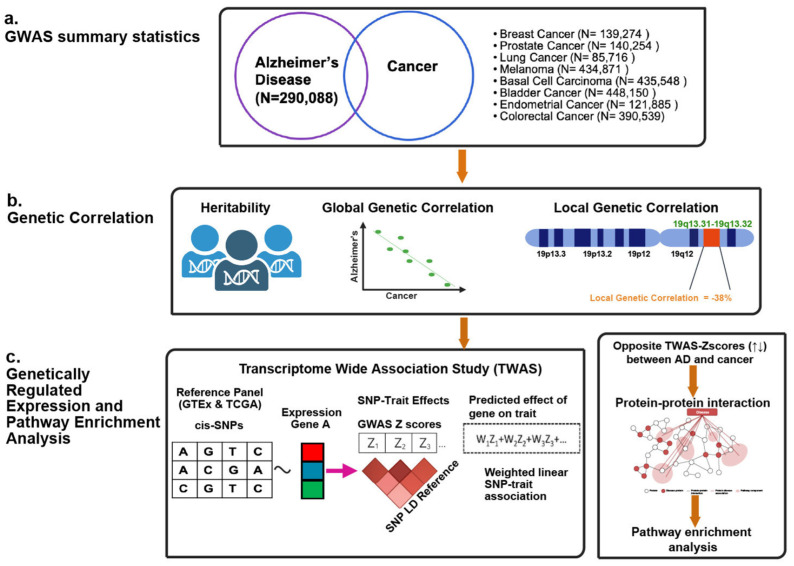
Overview of the experimental design. (**a**) **Summary of GWAS used for Alzheimer’s disease and cancers.** Venn diagram illustrating the GWAS sample sizes for Alzheimer’s disease (N = 290,088) and for each cancer types including breast cancer (N = 139,274), prostate cancer (N = 140,254), lung cancer (N = 85,716), melanoma (N = 434,871), basal cell carcinoma (N = 435,548), bladder cancer (N = 448,150), endometrial cancer (N = 121,885), and colorectal cancer (N = 390,539). All GWAS were based on European ancestry cohorts. (**b**) **Genetic correlation analysis between Alzheimer’s disease and cancers.** Schematic representation of heritability estimation, global and local genetic correlation estimation between Alzheimer’s disease and cancer phenotypes. (**c**) **Transcriptome-wide association study and functional analysis.** A schematic overview of the transcriptome-wide association study (TWAS) workflow integrating GWAS summary statistics, SNP–expression reference panels (e.g., GTEx), and linkage disequilibrium (LD) structure to predict genetically regulated expression and gene–trait association between Alzheimer’s disease and cancer phenotypes. Significant genes with inverse effect sizes, were further explored using protein–protein interaction networks and pathway enrichment analysis. Created in BioRender. Debnath, D. (2026) https://BioRender.com/09ivinh, accessed on 16 March 2026.

Therefore, these findings support the hypothesis that a small number of specific loci may drive the inverse risk relationship between AD and cancer, potentially through their effects on gene expression. Moreover, no prior study has examined local genetic correlations (rg) between AD and cancer. This study applies a novel framework by leveraging local rg and genetically regulated gene expression to investigate and help explain the inverse epidemiological patterns observed between the two diseases (Figure 1).

Genetically regulated gene expression (GReX) models estimate gene expression influenced by genetic variants by integrating GWAS findings with expression quantitative trait loci (eQTL) data [24]. This framework underlies transcriptome-wide association studies (TWAS), implemented here using FUSION (Functional Summary-based Imputation) [25]. Tissue-specific eQTL analyses are essential for interpreting genetic relationships, since the effects of regulatory variants can vary substantially across tissues [26]. To identify genes whose expression may contribute to the genetic correlations between AD and specific cancers, we leverage eQTL data from brain tissues relevant to AD and from tissues implicated in each cancer type. Furthermore, by examining pathways enriched among genes inversely associated with AD and cancer, we aim to highlight biological processes that are differentially regulated across the two diseases [27].

## 2. Results

### 2.1. SNP-Based Heritability Estimates (Observed Scale) and Global Genetic Correlation (rg) Estimates Between AD and Cancer Phenotypes

The observed scale heritability (h2) for AD (N = 290,088; Supplementary Table S1) and eight cancer types was significant (h2/SE; *p*-value < 0.05) (Supplementary Table S2), indicating statistical power for global genetic correlation. These findings are in line with earlier observed heritability studies of AD [18,28,29,30]. In assessing global genetic correlations between the meta-analyzed AD phenotype and various cancer phenotypes, most correlations were not statistically significant (*p* > 0.05), except for bladder cancer. (rg = −0.1854, *p* = 0.028) (Supplementary Table S3). We therefore tested whether specific genomic regions, rather than genome-wide effects, are correlated between AD and cancer types.

### 2.2. Local Genetic Correlation (rg) Between AD and Cancer Phenotypes Identifies Inversely Correlated Loci

**Figure 2:**
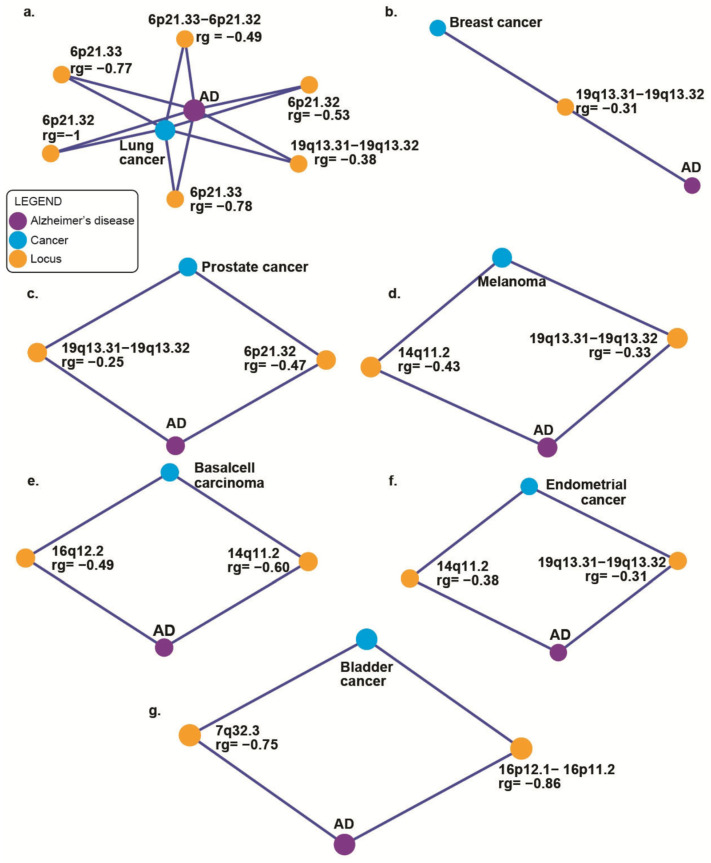
Local genetic correlation (rg) network graphs between Alzheimer’s disease and cancers. (**a**) **Local genetic correlation network between Alzheimer’s disease (AD) and lung cancer.** The network highlights significant local genetic correlation between Alzheimer’s disease and lung cancer within specific chromosomal regions. Each orange node signifies a genomic locus (cytoband), AD as purple, and cancer as blue nodes. The numbers on the edges indicate correlation coefficients (rg) between genomic regions or between a region and disease phenotype. (**b**) **Local genetic correlation network between Alzheimer’s disease (AD) and breast cancer.** The network highlights significant local genetic correlation between Alzheimer’s disease and breast cancer within specific chromosomal regions. Each orange node signifies a genomic locus (cytoband), AD as purple, and cancer as blue nodes.,. The numbers on the edges indicate correlation coefficients (rg) between genomic regions or between a region and disease phenotype. (**c**) **Local genetic correlation network between Alzheimer’s disease (AD) and prostate cancer.** The network highlights significant local genetic correlation between Alzheimer’s disease and prostate cancer within specific chromosomal regions. Each orange node signifies a genomic locus (cytoband), AD as purple, and cancer as blue nodes. The numbers on the edges indicate correlation coefficients (rg) between genomic regions or between a region and disease phenotype. (**d**) **Local genetic correlation network between Alzheimer’s disease (AD) and melanoma.** The network highlights significant local genetic correlation between Alzheimer’s disease and melanoma within specific chromosomal regions. Each orange node signifies a genomic locus (cytoband), AD as purple, and cancer as blue nodes. The numbers on the edges indicate correlation coefficients (rg) between genomic regions or between a region and disease phenotype. (**e**) **Local genetic correlation network between Alzheimer’s disease (AD) and Basal cell carcinoma.** The network highlights significant local genetic correlation between Alzheimer’s disease and basal cell carcinoma within specific chromosomal regions. Each orange node signifies a genomic locus (cytoband),AD as purple, and cancer as blue nodes. The numbers on the edges indicate correlation coefficients (rg) between genomic regions or between a region and disease phenotype. (**f**) **Local genetic correlation network between Alzheimer’s disease (AD) and endometrial cancer.** The network highlights significant local genetic correlation between Alzheimer’s disease and endometrial cancer within specific chromosomal regions. Each orange node signifies a genomic locus (cytoband), AD as purple, and cancer as blue nodes. The numbers on the edges indicate correlation coefficients (rg) between genomic regions or between a region and disease phenotype. (**g**) **Local genetic correlation network between Alzheimer’s disease (AD) and bladder cancer.** The network highlights significant local genetic correlation between Alzheimer’s disease and bladder cancer within specific chromosomal regions. Each orange node signifies a genomic locus (cytoband), AD as purple, and cancer as blue nodes. The numbers on the edges indicate correlation coefficients (rg) between genomic regions or between a region and disease phenotype.

Multiple genomic regions exhibited significant negative genetic correlations ((Figure 2) & (Supplementary Table S4)) between AD and different cancer phenotypes. The *APOE* locus (19q13.31–19q13.32) showed strong negative genetic correlations with several cancers, including breast (rg = −0.31, *p* = 0.036), prostate (rg = −0.25, *p* = 0.029), lung (rg = −0.38, *p* = 0.002), melanoma (rg = −0.33, *p* = 0.021), and endometrial cancer (rg = −0.31, *p* = 0.006). Likewise, the 14q11.2 region demonstrated significant negative genetic correlations with melanoma (rg = −0.43, *p* = 0.014), basal cell carcinoma (rg = −0.60, *p* = 0.001), and endometrial cancer (rg = −0.38, *p* = 0.008). For lung cancer, we found a cluster of five adjacent regions on chromosome 6 with strong negative genetic correlations with AD, with the strongest signals at 6p21.32 (rg = −1.00, *p* = 0.022) and 6p21.33 regions (rg = −0.78, *p* = 0.007). The major histocompatibility complex (MHC) is in this region of chromosome 6 [31]. We observed negative local genetic correlations between AD and bladder cancer at the 16p12.1–16p11.2 regions (rg = −0.86, *p* = 0.028) and 16q12.2 regions for basal cell carcinoma (rg = −0.49, *p* = 0.037). Given that we did not find any significant local genetic correlations between AD and colorectal cancer, we excluded it from follow-up analyses. We performed conditional analyses by deleting APOE4 variants (rs429358 and rs7412) from the GWAS summary statistics to determine whether the local genetic correlations were *APOE*-independent signals. We observed reduced and significant negative rg with lung cancer (rg = −0.31, *p* = 0.012) and insignificant negative local genetic correlations with breast (rg = −0.14, *p* = 0.334), prostate (rg = −0.08, *p* = 0.499), melanoma (rg = −0.21, *p* = 0.11), and endometrial cancer (rg = −0.17, *p* = 0.167) (Supplementary Table S4).

### 2.3. Transcriptome-Wide Association Study Reveals Shared and Divergent Genetic Architecture Between AD and Cancer Phenotypes

**Figure 3:**
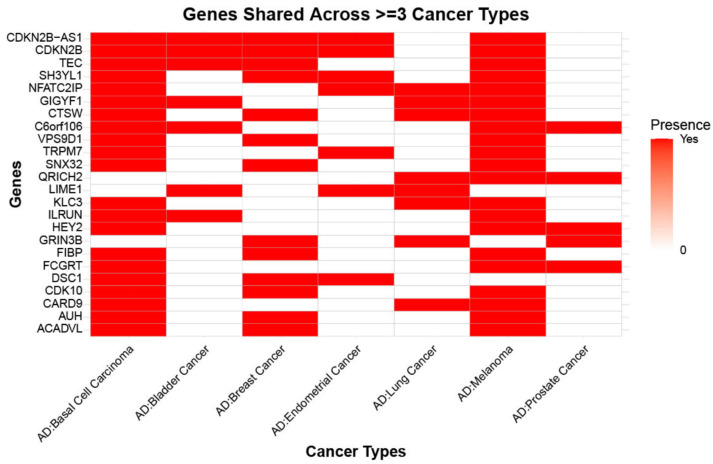
Genes shared between Alzheimer’s disease and multiple cancer types. The heatmap illustrates nominally significant genes that are commonly associated with Alzheimer’s disease and at least three cancer types. Rows represent genes, while columns represent AD–cancer type pairs. Red cells indicate the presence of a gene shared between AD and a given cancer type, whereas white cells represent absence of the shared genes.

To understand the role of genetically regulated gene expression, i.e., the role of eQTLs, we performed TWAS analysis using GWAS summary statistics for AD and seven cancer phenotypes (lung, breast, prostate, melanoma, basal cell carcinoma, bladder, and endometrial cancer) by the FUSION method [25]. We leveraged pre-computed gene expression weights from multiple tissue types, including brain tissue (DLPFC), cancer (TCGA), and disease-agnostic tissues (GTEx) [26,32,33]. We prioritized genes in each AD–cancer pair that were nominally significant in both AD and cancer (*p* < 0.05) and had inverse TWAS Z-scores (effect size), including lung cancer (60 genes), breast cancer (133 genes), prostate cancer (64 genes), melanoma (516 genes), basal cell carcinoma (550 genes), bladder cancer (69 genes), and endometrial cancer (43 genes) Our TWAS analysis revealed that 24 genes (Figure 3) had inverse effect size between three or more types of cancer and AD including *CDKN2B*, *TEC*, *SH3YL1*, *NFATC2IP*, *GIGYF1*, *CTSW*, *C6orf106*, *VPS9D1*, *TRPM7*, *SNX32*, *QRICH2*, *LIME1*, *KLC3*, *ILRUN*, *HEY2*, *GRIN3B*, *FIBP*, *FCGRT*, *DSC1*, *CDK10*, *CARD9*, *AUH*, and *ACADVL*.

### 2.4. AD and Lung Cancer

The TWAS analysis between AD and lung cancer (LC) (Supplementary Table S5) revealed 60 significant opposing directional genes, including 25 associations in DLPFC brain tissue, 12 in lung adenocarcinoma, 12 in lung squamous carcinoma, and 11 in lung tissue. We identified 8/60 significant genes that overlapped across multiple tissues (brain, lung adenocarcinoma, and lung squamous cell carcinoma), which include *AP4M1*, *CHRNA5*, *COG7*, *CTSW*, *DCTN5*, *EARS2*, *GATS*, and *MYST1* genes (Supplementary Figure S1a). The cholinergic receptor nicotinic alpha 5 subunit (*CHRNA5*) emerged as one of the most significant findings, showing inverse associations between AD and lung cancer across multiple tissues. In lung adenocarcinoma tissue, *CHRNA5* showed a modest positive association with (AD: Z = 2.36, *p* = 0.018) but a highly significant negative association with lung cancer (LC: Z = −15.72, *p* = 1.17 × 10^−55^). Similarly, in DLPFC brain tissue, *CHRNA5* exhibited a positive association with AD (AD: Z = 2.43, *p* = 0.015) while showing a strong negative association with lung cancer (LC: Z = −7.61, *p* = 2.70 × 10^−14^). *PSMA4* demonstrated the most significant lung cancer association identified in our analysis (LC: Z = 15.68, *p* = 1.93 × 10^−55^) in lung squamous carcinoma tissue, while showing a negative association with AD (AD: Z = −2.26, *p* = 0.024). Creatine kinase M-type (*CKM*) exhibited one of the strongest positive associations with AD in lung adenocarcinoma tissue (AD: Z = 8.65, *p* = 5.13 × 10^−18^) while showing a negative association with lung cancer (LC: Z = −2.78, *p* = 0.006). Our analysis revealed a region (19q13.31–32) of shared local genetic correlation between AD and lung cancer on chromosome 19, which contains two genes, including *CKM* and *KLC3*, with significant TWAS associations for both phenotypes (Supplementary Figure S2a).

### 2.5. AD and Breast Cancer

In AD and breast cancer (BC) TWAS analysis, we identified 133 (Supplementary Table S6) significant gene–trait associations across multiple tissues (40 associations identified in DLPFC brain tissue, 58 in breast mammary tissue, and 35 in breast invasive carcinoma). We identified 15 genes, including *ZNF404*, *EIF5A*, *TOM1L2*, *CYP51A1*, *MADD*, *CTSW*, *DDX55*, *DOK3*, *FIBP*, *FLVCR2*, *LRRC25*, *SNX32*, *TRIOBP*, *ZNF285*, and *MED13* (Supplementary Figure S1b) that showed significant associations across multiple tissue types. Among those genes that overlapped across multiple tissues were *ZNF404*, *EIF5A*, and *TOM1L2*. The *ZNF404* gene exhibited some of the most significant associations observed in our investigation, with similar patterns across all three tissue types, which was also previously reported in the breast tissue association [34]. In breast invasive carcinoma, *ZNF404* exhibited a strong negative association with AD (AD: Z = −3.75, *p* = 1.75 × 10^−4^) and a highly significant positive association with breast cancer (BC: Z = 7.49, *p* = 6.82 × 10^−14^). Similar patterns were observed in DLPFC brain tissue (AD: Z = −3.40, *p* = 6.69 × 10^−4^; BC: Z = 7.59, *p* = 3.13 × 10^−14^) and breast mammary tissue (AD: Z = −3.33, *p* = 8.63 × 10^−4^; BC: Z = 7.60, *p* = 3.01 × 10^−14^). *LRRC25* is very consistent across tissues and has significant negative links to AD and positive links to breast cancer (AD: Z = −3.93, *p* = 8.67 × 10^−5^) and (BC: Z = 7.62, *p* = 2.60 × 10^−14^), with even stronger associations in breast mammary tissue (AD: Z = −5.43, *p* = 5.77 × 10^−8^; BC: Z = 8.01, *p* = 1.13 × 10^−15^). Similarly, *SSBP4* showed a strong association with breast cancer in breast mammary tissue (BC: Z = 11.00, *p* = 3.83 × 10^−28^), while maintaining a negative association with AD (AD: Z = −3.74, *p* = 1.81 × 10^−4^). We observed a local genetic correlation between AD and breast cancer in the 19q13.31–19q13.32 region and also demonstrated significant TWAS associations. (Supplementary Figure S2b). These included metabolic and regulatory genes such as *ETHE1* [35], *PHLDB3* [36], several zinc finger protein genes with potential transcriptional regulatory functions (such as *ZNF221*, *ZNF285*, and *ZNF404*), and *LYPD5* [37].

### 2.6. AD and Prostate Cancer

We observed 64 genes across DLPFC brain tissue, prostate tissue, and prostate cancer tissue (Supplementary Table S7) that were significantly associated with both AD and prostate cancer (PC) and had inverse effects. Among the 64 observed genes, 24 showed significant associations in DLPFC brain tissue, 19 in prostate tissue, and 21 in prostate adenocarcinoma samples. We looked at genes exhibiting notable signals in many tissue types to find genes with strong cross-tissue correlations. Five genes demonstrated significant associations across at least two tissue types: *FOLH1*, *PTRH1*, *C6orf57*, *ZNF285*, and *TBX1* (Supplementary Figure S1c). One significant finding is the association of *FOLH1*, which encodes prostate-specific membrane antigen (PSMA), with the risk of both AD and prostate cancer [38]. In DLPFC tissue, *FOLH1* demonstrated a strong positive association with AD risk (AD: Z = 3.545, *p* = 3.93 × 10^−4^) and an inverse association with prostate cancer risk (PC: Z = −2.871, *p* = 0.004). This pattern was replicated in prostate adenocarcinoma tissue (AD: Z = 3.673, *p* = 2.4 × 10^−4^; PC: Z = −2.202, *p* = 0.028). Several genes involved in transcriptional regulation showed significant associations across tissues. *TOM1L2* exhibited one of the strongest associations in DLPFC tissue (AD: Z = 3.273, *p* = 1.07 × 10^−3^; PC: Z = −4.653, *p* = 3.28 × 10^−6^), suggesting its role as a key regulatory node [39]. The transcription factor *TBX1* demonstrated particularly striking associations in prostate tissue (AD: Z = 2.552, *p* = 0.011; PC: Z = −6.829, *p* = 8.55 × 10^−12^) and prostate adenocarcinoma (AD: Z = 2.044, *p* = 0.04096; PC: Z = −4.662, *p* = 3.14 × 10^−6^), representing one of the strongest effect sizes observed in our analysis [40]. Our investigation showed that AD and prostate cancer have a local genetic link in chromosome 19 (cytoband 19q13.31–13.32), an area that also showed strong TWAS associations. Combining the TWAS data with the local genetic correlation results showed that *ZNF285* (19q13.31) gene is in this chromosomal region (Supplementary Figure S2c).

### 2.7. AD and Melanoma

We identified 516 genes (Supplementary Table S8) demonstrating significant associations with both AD and melanoma (ML) risk across multiple tissue types. This analysis revealed extensive tissue-specific expression patterns, with the DLPFC brain tissue harboring 50 genes, skin tissues (including transformed fibroblasts, sun-exposed, and non-sun-exposed) collectively representing 289 genes, and adipose tissues containing 175 genes. *HEY2*, encoding a basic helix-loop-helix transcription factor, demonstrated strong inverse associations in DLPFC (AD: Z = −3.231, *p* = 0.001; ML: Z = 2.102, *p* = 0.036) and skin-transformed fibroblasts (AD: Z = −3.126, *p* = 0.002; ML: Z = 2.226, *p* = 0.026), implicating notch signaling pathway dysregulation in both conditions [41]. A large, shared susceptibility locus was identified in the chromosome 17q21 region, which included 103 genes with significant correlations. *MAPT*, encoding the tau protein important to AD pathology, demonstrated different associations in DLPFC (AD: Z = 3.915, *p* = 9.04 × 10^−5^; ML: Z = −2.900, *p* = 0.004) and skin tissues [42]. Multiple pseudogenes, *LRRC37A* family members, *KANSL1*, and *CRHR1* are co-localized in this region, indicating common regulatory mechanisms influencing melanoma susceptibility and neurodegeneration [43]. *ASIP*, an MC1R antagonist, showed an extraordinary melanoma association in sun-exposed skin (ML: Z = 25.347, *p* = 9.61 × 10^−142^), the strongest signal observed across all genes, while maintaining a modest inverse AD association (AD: Z = −2.006, *p* = 0.045) [44]. *CDK10* had the most significant opposite effects in different tissues. It had positive AD connections and very significant negative melanoma associations in adipose (ML: Z = −27.857, *p* = 8.87 × 10^−171^) and skin tissues (ML: Z = −30.347, *p* = 2.77 × 10^−202^). *CDKN2B*, encoding the tumor suppressor p15INK4b, showed significant associations in DLPFC (AD: Z = 3.618, *p* = 2.96 × 10^−4^; ML: Z = −6.743, *p* = 1.56 × 10^−11^), highlighting the inverse relationship between cell cycle inhibition in neurodegeneration versus melanoma. Several genes involved in immune regulation demonstrated significant associations. *MS4A2*, encoding the high-affinity IgE receptor beta chain [45], showed extreme opposing effects in adipose tissue (AD: Z = −9.962, *p* = 2.24 × 10^−23^; ML: Z = 2.328, *p* = 0.020) and skin (AD: Z = −7.995, *p* = 1.29 × 10^−15^; ML: Z = 2.624, *p* = 0.009). Twenty-four genes showed significant associations across at least two tissue types including DLPFC brain tissue: *ANKH*, *ARHGAP27*, *CAPN10*, *CDKN2B*, *CPNE7*, *DAGLB*, *EGR2*, *FIBP*, *FOLH1*, *GEMIN7*, *HEY2*, *KLC3*, *LRRC37A2*, *MAPT*, *NFATC2IP*, *NSF*, *ORMDL3*, *POLR2E*, *QPCT*, *RBM6*, *SNX32*, *TRPM7*, *VPS33A*, and *ZCRB1* (Supplementary Figure S1d). Through combined TWAS and local genetic correlation analysis, we found a shared genetic correlation between AD and melanoma on the chromosome 19q13.31–19q13.32 genomic region (Supplementary Figure S2d). We discovered 5 genes, *CEACAM19*, *GEMIN7*, *ETHE1*, *RTN2*, and *KLC3*, that show strong relationships across many tissues, suggesting that this area shares genetic architecture with AD and melanoma risk factors.

### 2.8. AD and Basal Cell Carcinoma

We identified 550 genes (Supplementary Table S9) associated with both AD and basal cell carcinoma (BCC) across multiple tissue types. This study discovered tissue-specific expression patterns for genes in the brain, skin, and adipose tissues, with 45, 310, and 195 genes, respectively. Notably, 17 of the 45 genes from DLPFC brain tissue showed significant associations with peripheral tissues, indicating systemic links between neurodegeneration and basal cell carcinoma susceptibility. Eighty genes showed significant associations across at least two tissue types (Supplementary Figure S1e). *CPNE7* demonstrated the opposing effects, with DLPFC showing (AD: Z = −2.174, *p* = 0.030) and (BCC: Z = 8.892, *p* = 5.99 × 10^−19^), while skin-transformed fibroblasts exhibited (AD: Z = 2.682, *p* = 0.007) and (BCC: Z = −28.299, *p* = 3.56 × 10^−176^). *MAPT* displayed opposing effects in DLPFC (AD: Z = −3.915, *p* = 9.04 × 10^−5^; BCC: Z = −5.052, *p* = 4.37 × 10^−7^) and Skin-Not Sun Exposed (Suprapubic) (AD: Z = −3.813, *p* = 1.37 × 10^−4^; BCC: Z = 5.065, *p* = 4.09 × 10^−7^). *CENPBD1* showed opposing effects in DLPFC (AD: Z = −2.639, *p* = 0.008; BCC: Z = 8.711, *p* = 3.00 × 10^−18^), with corresponding strong signals in adipose tissues (visceral adipose: AD: Z = −3.243, *p* = 0.001; BCC: Z = 7.210, *p* = 5.59 × 10^−13^). *GEMIN7* showed the strongest cross-tissue pattern with associations in DLPFC (AD: Z = 3.754, *p* = 1.74 × 10^−4^; BCC: Z = −2.518, *p* = 0.012) and consistent signals across adipose tissues (subcutaneous: AD: Z = 7.845, *p* = 4.33 × 10^−15^; BCC: Z = −1.976, *p* = 0.048; visceral: AD: Z = 7.636, *p* = 2.24 × 10^−14^; BCC: Z = −2.170, *p* = 0.03). The 17q21 chromosomal region became a large, shared susceptibility locus, with 106 genes with notable correlations. This region’s complex inversion polymorphism affects multiple genes, including *MAPT*, *KANSL1*, *CRHR1*, and the *LRRC37A* family, all showing strong inverse AD-BCC associations [43]. *CDK10* exhibited the most extreme BCC associations across all tissues, with Z-scores reaching −23.4 (*p* = 6.78 × 10^−121^) in skin tissues while maintaining positive AD associations (AD: Z = 2.451, *p* = 0.014). *CDKN2B*, encoding p15INK4b tumor suppressor [46], showed extraordinary associations in DLPFC (AD: Z = 3.618, *p* = 2.96 × 10^−4^; BCC: Z = −12.7, *p* = 8.41 × 10^−37^), representing one of the strongest signals in our analysis. Multiple DNA repair genes showed significant associations. *ERCC2*, essential for nucleotide excision repair [47], demonstrated strong associations particularly in skin tissues (sun-exposed: AD: Z = 8.6, *p* = 7.76 × 10^−18^; BCC: Z = −2.73, *p* = 0.006), suggesting differential DNA damage response between neurodegeneration and skin carcinogenesis. *MS4A2*, encoding the high-affinity IgE receptor beta chain [48], showed extreme opposing effects in skin tissue (AD: Z = −7.995, *p* = 1.29 × 10^−15^; BCC: Z = 2.424, *p* = 0.015), suggesting differential allergic and inflammatory responses between AD and BCC.

### 2.9. AD and Endometrial Cancer

We found 43 genes (Supplementary Table S10) with significant associations for both AD and endometrial cancer (EC) across multiple tissue types. This study revealed tissue-specific expression patterns with 23 genes identified in DLPFC brain tissue, 17 genes in uterus tissue, and 3 genes in uterine endometrial carcinoma tissue. Among the 23 genes identified in DLPFC brain tissue, 2 genes showed significant associations with uterine and uterus tissues, indicating shared genetic mechanisms between neurodegeneration and endometrial cancer susceptibility (Supplementary Figure S1f). *TIMM10* showed the most consistent cross-tissue pattern, with significant associations in DLPFC (AD: Z = −2.113, *p* = 0.035; EC: Z = 2.266, *p* = 0.024), uterine endometrial carcinoma tissue (AD: Z = −2.803, *p* = 0.005; EC: Z = 2.427, *p* = 0.015), and normal uterus tissue (AD: Z = −2.017, *p* = 0.044; EC: Z = 2.375, *p* = 0.018). *INTS1* showed significant associations in both DLPFC (AD: Z = −2.045, *p* = 0.041; EC: Z = 2.371, *p* = 0.018) and uterus tissue (AD: Z = −2.640, *p* = 0.008; EC: Z = 2.070, *p* = 0.039), implicating RNA processing dysfunction as a shared mechanism [49]. *CDKN2B* and *CDKN2B-AS1* at the 9p21 locus showed remarkable associations in DLPFC. *CDKN2B* demonstrated (AD: Z = 3.618, *p* = 2.96 × 10^−4^) and (EC: Z = −3.308, *p* = 9.38 × 10^−4^), while *CDKN2B-AS1* showed (AD: Z = 2.160, *p* = 0.031) and (EC: Z = −3.436, *p* = 5.9 × 10^−4^). These tumor suppressor genes’ opposing effects suggest differential cell cycle regulation between neurons and endometrial cells [46]. *SNX1* showed the strongest AD association in DLPFC (AD: Z = −5.132, *p* = 2.87 × 10^−7^; EC: Z = 2.358, *p* = 0.018), implicating endosomal protein sorting dysfunction. *SH3YL1* in uterine endometrial carcinoma tissue showed strong opposing effects (AD: Z = 3.195, *p* = 0.001; EC: Z = −2.652, *p* = 0.008), suggesting roles in cellular signaling and cytoskeletal organization. *LAT* demonstrated strong EC association in uterus tissue (AD: Z = −2.420, *p* = 0.016; EC: Z = 3.640, *p* = 2.73 × 10^−4^), highlighting immune system involvement.

### 2.10. AD and Bladder Cancer

Our investigation revealed 69 significant gene–trait associations (Supplementary Table S11) between AD and bladder cancer (BLC) across three distinct tissue sources, which includes 38 genes in whole blood tissue, 24 genes in DLPFC brain tissue, and 7 genes in bladder urothelial carcinoma tissue. Notably, five genes (Supplementary Figure S1g) exhibited cross-tissue associations with consistent expression patterns across multiple tissue types, which was the most remarkable discovery. These cross-tissue genes included *EARS2*, *NDE1*, *SUPT5H*, *TAS2R4*, and *TEC*. The cross-tissue expression profile was observed by *EARS2*, which had associations with each of the three tissue types that were studied. In bladder urothelial carcinoma tissue, *EARS2* exhibited a positive association with AD risk (AD: Z = 2.674, *p* = 0.008) and a negative association with bladder cancer risk (BLC: Z = −2.454, *p* = 0.014). Similar directional patterns were observed in DLPFC brain tissue (AD: Z = 2.664, *p* = 0.008; BLC: Z= −2.173, *p* = 0.030) and whole blood tissue (AD: Z = 2.855, *p* = 0.004; BLC: Z = −2.159, *p* = 0.031). *SUPT5H* exhibited intriguing tissue-specific directional effects. In whole blood tissue, the gene showed a positive association with AD (AD: Z = 2.06, *p* = 0.039) and a negative association with bladder cancer (BLC: Z = −2.522, *p* = 0.012). Conversely, in both DLPFC brain tissue and bladder urothelial carcinoma tissue, *SUPT5H* demonstrated negative associations with AD and positive associations with bladder cancer, suggesting tissue-specific regulatory mechanisms that may differentially impact disease susceptibility. The AD-BLC link was highlighted by 24 significant gene correlations in DLPFC brain tissue. Notable genes included *CDKN2B* (AD: Z = 3.618, *p* = 2.96 × 10^−4^), a tumor suppressor gene previously implicated in melanoma and basal cell carcinoma and neurodegeneration, and *CST3* (AD: Z = 2.919, *p* = 0.004 for AD), encoding cystatin C, a well-established AD biomarker [50]. The most significant AD association was observed for *MS4A6A* (AD: Z = 9.496, *p* = 2.18 × 10^−21^), a well-established AD susceptibility gene located in the *MS4A* gene cluster on chromosome 11 [51]. Interestingly, *MS4A6A* also demonstrated a negative association with bladder cancer risk (BLC: Z = −2.740, *p* = 0.006), suggesting a potential protective effect against bladder cancer development.

Additionally, for the genes observed on locus 19q13, we excluded the *APOE* variants (rs429358 and rs7412), to determine whether the TWAS signals on chromosome 19 were *APOE* independent. After this exclusion, the same genes remained significant and inverse expression between AD and breast, prostate, lung, and melanoma cancers (Supplementary Tables S5–S8).

### 2.11. Pathway Enrichment of Inversely Associated Genes Between AD and Cancer Based on Their Protein–Protein Interactions

To prioritize the functional role of genes that are inversely expressed between each cancer type and AD, we identified the protein–protein interaction (PPI) network from IMEx database [52]. We performed comprehensive pathway enrichment analysis to explore the biological processes linking AD and various cancers. For genes inversely regulated between AD and lung cancer, several pathways showed significant enrichment. The most strongly associated pathway was the epidermal growth factor receptor signaling pathway (*p* = 1.30 × 10^−5^). Additional significant pathways included the mitotic cell cycle checkpoint (*p* = 1.01 × 10^−4^), regulation of the mitotic cell cycle (*p* = 1.03 × 10^−4^), and the cell cycle checkpoint pathway (*p* = 2.35 × 10^−4^) (Supplementary Table S12).

For genes inversely regulated between AD and breast cancer, the most strongly associated pathway was the transforming growth factor beta receptor signaling pathway (*p* = 1.12 × 10^−6^). Additional significant pathways included the transmembrane receptor protein serine/threonine kinase signaling pathway (*p* = 4.89 × 10^−6^), regulation of protein metabolic processes (*p* = 8.82 × 10^−5^), and positive regulation of metabolic processes (*p* = 1.04 × 10^−4^) (Supplementary Table S13).

For genes inversely regulated between AD and prostate cancer, the most strongly associated pathway was regulation of the apoptotic process (*p* = 1.77 × 10^−9^). Additional significant pathways included positive regulation of transcription from the RNA polymerase II promoter (*p* = 1.63 × 10^−7^), programmed cell death (*p* = 2.38 × 10^−7^), and positive regulation of transcription, DNA-dependent (*p* = 4.03 × 10^−7^) (Supplementary Table S14).

For genes inversely regulated between AD and melanoma, several pathways showed strong and highly significant enrichment. The most significant pathway was signal transduction in response to DNA damage (*p* = 8.82 × 10^−11^). Additional enriched pathways included regulation of the apoptotic process (*p* = 3.69 × 10^−10^) and the DNA damage response mediated by p53 class signaling (*p* = 1.11 × 10^−8^) (Supplementary Table S15).

For genes inversely regulated between AD and basal cell carcinoma, several pathways showed significant enrichment. The most strongly associated pathway was blood coagulation (*p* = 1.15 × 10^−9^). Additional enriched pathways included interphase of the mitotic cell cycle (*p* = 5.78 × 10^−8^), response to wounding (*p* = 6.30 × 10^−8^), regulation of programmed cell death (*p* = 1.50 × 10^−7^), and the innate immune response (*p* = 2.90 × 10^−7^) (Supplementary Table S16).

For genes inversely regulated between AD and endometrial cancer, one pathway reached statistical significance: regulation of the mitotic cell cycle (*p* = 5.02 × 10^−5^) (Supplementary Table S17).

For genes inversely regulated between AD and bladder cancer, several pathways showed significant enrichment. The most strongly associated pathway was negative regulation of the cell cycle (*p* = 4.50 × 10^−6^). Additional enriched pathways included negative regulation of transcription, DNA-dependent (*p* = 1.93 × 10^−5^) and negative regulation of metabolic processes (*p* = 3.60 × 10^−5^) (Supplementary Table S18).

## 3. Discussion

Building on prior evidence of inverse comorbidity between AD and cancer, earlier genetic studies have largely relied on genome-wide approaches such as global genetic correlation and Mendelian randomization [53]. To deepen mechanistic understanding, this study shifts focus toward determining whether specific genomic regions, rather than broad genome-wide effects, are driving the observed inverse relationship. Our investigation provides further molecular support of the previously reported inverse epidemiological association between AD and certain cancers by identifying significant negative rg within regions on chromosomes 6, 14, and 19. The genomic region with the greatest number of observations was chromosome 19q13.31–19q13.32, which showed significant negative genetic correlations with the highest number of cancer types, including breast, prostate, lung, melanoma, and endometrial cancer. After removing variants in the *APOE* locus, the local genetic correlation in the 19q13 region remained significant for only lung cancer. This indicates that the shared genetic signal between several cancers was being driven by the *APOE* SNPs. The apolipoprotein E (*APOE*) ε4 carries the highest genetic risk for late-onset AD [23,54,55,56]. The three main forms of *APOE* (ε2, ε3, and ε4) differently affect neuroinflammation, tau pathology, Aβ aggregation, clearance, cerebrovascular stability, and lipid transport [56,57]. *APOE* promotes tumor development by enhancing immune suppression, affecting the tumor microenvironment through NF-κB signaling and lipid receptor interactions (LDLR, LRP8), and facilitating cell proliferation, migration, and metastasis [58,59]. Tumor-associated macrophages (TAMs) are a major cellular source of *APOE* in the tumor microenvironment, serving as a tumor biomarker for lung cancer [60], and colorectal cancer [61]. Although in certain cancers such as ovarian cancer and melanoma, it exerts protective effects [62,63]. This finding aligns with the well-established role of *APOE* as the primary genetic susceptibility locus for late-onset AD, suggesting that genetic variants in this region may exert opposing effects on AD risk and cancer susceptibility [23,64,65,66,67].

However, additional genes in this region suggest variants/genes beyond *APOE* contribute to the AD–cancer inverse relationship. Recent studies have identified *APOE*-independent genetic variants in this region that influence AD risk and tau levels [68]. The consistent association of this chromosomal region across multiple cancer types supports shared genetic mechanisms underlying the inverse epidemiological relationship between AD and cancer [9]. Notably, no significant genetic correlation was observed between AD and colorectal cancer, indicating that the inverse relationship may not be universal across all cancer types. The 19q13 region includes non-coding regulatory elements, and recent epigenomic profiling has demonstrated that AD-associated variants in this locus fall predominantly within enhancers and open chromatin regions active in microglia and astrocytes rather than neurons [69], suggesting that glial cell-type-specific regulatory mechanisms drive a substantial component of the observed genetic signal. Critically, microglial enhancer activity at this locus overlaps with regulatory elements also implicated in TAMs function, provides a plausible shared immune-regulatory mechanism underlying pleiotropy between AD and cancer [70]. Furthermore, non-coding variants in *TOMM40*, *APOC1*, and *PVRL2*, through long-range chromatin interactions that are highly cell-type-dependent [71], promote neurodegeneration in microglia while influencing proliferative or immune-evasion pathways in tumor microenvironments.

This result is consistent with prior epidemiological research that has shown negative relationships between AD and other cancers, although the strength of these connections varies across cancer types [12]. The concept of “antagonistic pleiotropy” offers another framework for comprehending how evolutionary selection may have influenced genetic variants having contradictory effects on various disease outcomes [13].

Our comprehensive TWAS analysis investigated genes associated with AD and seven cancer types across disease-agnostic brain and relevant tissues, as well as cancer-specific tissues, revealing significant shared genomic architecture with some tissue-specific expression patterns [27,72,73,74,75,76,77]. We identified 24 nominally significant genes expressed in at least three cancer types associated with AD transcriptome-wide analysis. *CHRNA5*, a gene encoding the neuronal nicotinic acetylcholine receptor α5 subunit, was the top candidate gene showing opposing relationships between lung adenocarcinoma and AD. *CHRNA5* expression increased in DLPFC brain tissue but decreased in lung adenocarcinoma. This aligns with previous research showing *CHRNA5* on chromosome 15q25 (particularly rs16969968 and rs12914385) increases lung cancer risk through nicotine dependency pathways [78,79]. In contrast, recent neuropathological research correlates elevated *CHRNA5* expression with reduced Aβ accumulation in human prefrontal cortex, indicating a neuroprotective role in AD [80]. We found that lower predicted expression of *PSMA4* in DLPFC tissue might be associated with higher AD risk. *PSMA4*, a proteasome complex component, assists misfolded protein clearance and is implicated in AD. Consistent with other studies showing decreased *PSMA4* expression in AD brain tissue, our results suggest impaired proteasomal function contributes to toxic Aβ and tau protein accumulation and neurodegeneration in AD [81,82]. Higher *PSMA4* expression was significantly associated with increased lung squamous carcinoma risk, consistent with previous studies linking the 15q25 locus (*CHRNA5*-*CHRNA3*-*PSMA4* cluster) to lung cancer susceptibility [78,79]. *PSMA4* encodes a 20S proteasome subunit critical for protein degradation and cell cycle regulation, playing a key role in cancer development and progression. Elevated *PSMA4* levels correlate with poor prognosis, chemotherapy resistance, and increased cancer cell proliferation in multiple malignancies, including multiple myeloma, lung, and breast cancers [83].

We identified that *CDKN2B-AS1* gene expression in DLPFC brain tissue was upregulated in AD and downregulated in breast, melanoma, basal cell carcinoma, endometrial, and bladder cancer. The antisense transcript *CDKN2B-AS1* is located at the 9p21 locus and is known to influence the nearby tumor suppressor gene *CDKN2B*, which governs the progression of the cell cycle. Abnormal regulation of this locus has been linked to AD and several types of malignancies [84,85]. In brain tissue, TWAS analysis revealed that *FCGRT* at 19q13.33 was downregulated in prostate cancer but upregulated in AD, melanoma, and basal cell carcinoma. *FCGRT* encodes the neonatal Fc receptor (FcRn), which regulates IgG recycling and immune surveillance. Increased FcRn in the brain may enhance Aβ clearance through improved IgG trafficking but also triggers chronic neuroinflammation, contributing to AD pathogenesis [86]. However, in cancer, decreased FcRn expression aids tumor growth by preventing the transport of antigens and the recycling of IgG, which enables cancer cells to avoid immune monitoring as well as changes albumin turnover, which in turn increases food availability, which promotes tumor development and metabolic reprogramming [87]. *HEY2*, a Notch signaling transcription factor, is downregulated in AD brain tissue but upregulated in prostate cancer, melanoma, and basal cell carcinoma. In the brain, Notch signaling, along with its downstream effectors *HEY1*/*HEY2*, regulates blood–brain barrier integrity, vascular stability, and neuronal development. Disrupted Notch-*HEY2* signaling may exacerbate AD-related neurodegeneration and age-related vascular degeneration [88]. In cancer, *HEY2* promotes tumor angiogenesis and vascular formation. Dysregulated *HEY2* expression in prostate and other malignancies is linked to enhanced tumor vascularization and progression. *HEY2* may also facilitate invasion and metastasis by regulating tumor suppressor pathways and promoting epithelial-to-mesenchymal transition via TGF-β/Smad signaling. These results suggest that reduced *HEY2* in the brain aggravates AD neurodegeneration and vascular impairments, while elevated *HEY2* in prostate tissue drives angiogenic and carcinogenic processes.

*CDK10*, a transcriptional and cell cycle regulator, is upregulated in skin and adipose tissues in AD but downregulated in the same tissues in melanoma and basal cell carcinoma. Similarly, *CDK10* is upregulated in breast tissue in AD but downregulated in breast cancer. *CDK10* regulates neuronal cell cycle re-entry and the modulation of transcriptional programs. Elevated *CDK10* in adipose tissue may reflect systemic changes affecting neuronal cell cycle dynamics in AD. Studies show *CDK10* expression is significantly higher in AD patients, especially in the cerebellum. It may also worsen the neuronal death and dysfunction that characterizes AD, suggesting its potential as a diagnostic biomarker [89,90]. The tumor suppressor roles played by *CDK10* in cancer are tissue-specific. It regulates transcription, proliferation, cell cycle, and drug sensitivity. In breast, hepatic, and biliary cancers, *CDK10* overexpression inhibits tumor growth and enhances treatment sensitivity. Conversely, in colorectal cancer, it acts as an oncogene, promoting tumor development and reducing survival, indicating potential therapeutic value [91,92]. *TRPM7*, a channel kinase regulating cellular signaling and magnesium homeostasis, is upregulated in skin-derived transformed fibroblasts in melanoma but downregulated in AD brain tissue (melanoma, basal cell carcinoma, and endometrial cancer). The kinase activity of *TRPM7* is essential for Aβ clearance, a key feature of AD pathogenesis. Studies show *TRPM7* is downregulated in AD hippocampal tissue, and increasing its levels may prevent Aβ-induced synapse loss, suggesting that reduced brain *TRPM7* impairs Aβ clearance and accelerates neurodegeneration [93,94]. Melanoma formation has been associated with *TRPM7*, whose expression levels influence tumor behavior. In cancer, *TRPM7* plays a complex, potentially oncogenic role by regulating calcium and magnesium levels, promoting tumor proliferation, invasion, and migration. Elevated *TRPM7* expression correlates with poor prognosis and metastasis across multiple cancers, including melanoma, making it a potential therapeutic target [95,96].

We identified significant negative local rgs between AD and several cancers (lung, breast, prostate, melanoma, and endometrial cancer) in the 19q13.31–19q13.32 genomic region. TWAS analysis revealed shared genes including *CKM* and *KLC3* (lung cancer), *ETHE1*, *LYPD5*, *PHLDB3*, *ZNF221*, *ZNF285*, and *ZNF404* (breast cancer), *ZNF285* (prostate cancer), and *CEACAM19*, *ETHE1*, *GEMIN7*, *KLC3*, and *RTN2* (melanoma) within this locus. Previous studies show neighboring genes (*TOMM40*, *APOC1*) in the 19q13.31–19q13.32 cluster contain regulatory variants that independently or jointly modify AD risk by various mechanisms beyond *APOE* ε-alleles [71]. Despite *APOE* being the traditional Alzheimer’s risk locus at 19q13.31–19q13.32, our TWAS discovered signals across all cancer types associated with AD that extended throughout the wider 19q13 band (sub-bands 19q13.11 → 19q13.41). We identified *LRRC25* (19q13.11), *SIGLEC11* (19q13.33), *ZNF528-AS1*, and *ZNF880* (19q13.41) for breast cancer; *FCGRT* (19q13.33) and *SUPT5H* (19q13.2) for prostate cancer; *CEP89* (19q13.11), *FCGRT* (19q13.33), and *ZNF415* (19q13.42) for melanoma; and *FBXO17* (19q13.2) for endometrial cancer. Despite the exclusion of *APOE* variants, TWAS identified the same genes in the 19q13 region associated with AD in lung, breast, prostate, and melanoma, including *CKM* and *KLC3*. This is likely related to the strong LD and prevalent regulatory architecture within the *APOE* haplotype block. The remaining variants exhibit regulation of genes in the region. This observation likely indicates the role of genes in this region participating in pathways influencing both neurodegeneration and tumorigenesis in tissue-dependent ways, including lipid/vesicle trafficking (*FCGRT*) and immune regulation (*LRRC25*, *SIGLEC11*, *MS4A* family).

**Figure 4:**
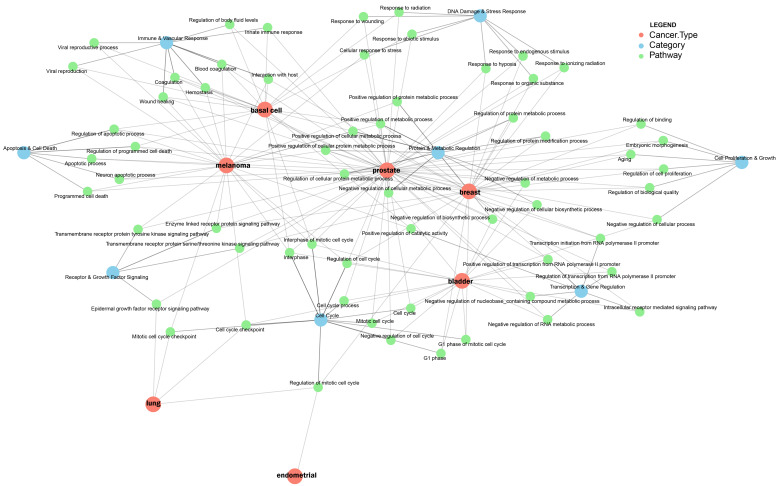
Pathways shared between Alzheimer’s disease and multiple cancer types. The network plot shows pathways (green circles) shared across two or more cancer types (red circles) grouped by major category (blue circles) and connected with edges across cancer.

Across cancer types, genes inversely regulated with AD consistently converged on pathways involved in cell-cycle control, apoptosis, DNA-damage signaling, and metabolic regulation (Figure 4). These shared patterns suggest that the biological processes promoting uncontrolled proliferation and survival in cancer may oppose those contributing to neurodegeneration in AD. The repeated enrichment of pathways related to p53 signaling, programmed cell death, and transcriptional regulation further supports a model in which core regulatory networks are differentially activated across the two diseases. Together, these findings underscore a set of mechanistic axes, i.e., cell-cycle dynamics, stress-response signaling, and immune or tissue-repair pathways. Regarding the cell cycle, oncoproteins such as cyclins D1 and B1 and CDK4/6, which normally control cell proliferation, are discovered to abnormally reactivate in post-mitotic AD neurons. This causes them to enter an incomplete cell cycle, which ends in apoptosis instead of division. This process occurs before tau tangle and amyloid plaque formation [97,98]. Another significant area where cancer and AD intersect is immune system dysfunction. Inhibitory immune checkpoint receptors, such as PD-1, SIRPα, and SIGLEC family members, have been shown to be upregulated by microglia in the AD brain. Tumors also use these receptors to avoid immune surveillance. Persistent neuroinflammation may be caused by the activation of these checkpoint mechanisms, which may prevent microglial clearance of Aβ [99]. At the level of genome integrity, Aβ-driven proteasomal degradation preferentially depletes *BRCA1*, a well-known tumor suppressor essential to DNA double-strand break repair, in AD neurons. This results in the buildup of unrepaired DNA lesions and increasing synaptic dysfunction [100]. This opposing dysregulation is further demonstrated by the PI3K/AKT/mTOR signaling axis, which is constitutively activated in cancer to maintain cell survival and proliferation. In AD neurons, however, the same pathway facilitates aberrant cell cycle re-entry, suppresses autophagy-mediated protein clearance, and promotes tau hyperphosphorylation via CDK5, all of which accelerate neuronal death [101]. Lastly, both diseases are caused by disrupted proteostasis, which is maintained by the ubiquitin-proteasome system and autophagy. However, the mechanisms by which these systems fail are different. In cancer cells, for example, chaperones like HSP90 are upregulated, and the proteasomal capacity is enhanced to handle the stress of rapid proliferation. In AD, on the other hand, neurons gradually lose their ability to handle the degradation of Aβ and tau aggregates, leading to proteotoxic cell death [102]. Vascular regulation such as angiogenesis is actively and persistently upregulated to sustain tumor growth, whereas in AD, vascular changes are stage-dependent, i.e., early compensatory increases in cerebral blood flow and angiogenic signaling eventually give way to progressive hypoperfusion and reduced VEGF expression in later stages [103]. Together, these results indicate the biological pathways that may underlie the long-observed inverse epidemiological relationship between AD and multiple cancers.

This research has several limitations that should be noted. First, we only included GWAS summary results from cohorts with predominantly European ancestry in our analysis, as GWAS in other ancestries are either not available or severely underpowered. This reduces confounding from population stratification but may restrict the applicability of our results to non-European ancestries, where allele frequencies and linkage disequilibrium patterns vary considerably. Secondly, although local genetic correlation and TWAS provide significant insights into the shared genomic architecture and GReX, they are unable to establish direct causal links between AD and cancer risk. Additionally, TWAS models rely on the tissue relevance and quality of reference eQTL panels, which we aimed to account for where possible. Importantly, our analysis leveraged both disease-agnostic GTEx v8 reference panels and cancer-specific TCGA expression panels, which strengthens our findings by capturing both general regulatory architecture and tumor-relevant gene expression contexts; however, they are not comprehensive enough to capture context-specific regulatory effects. TWAS models vertical pleiotropy combining variant mediating risk on disease via regulating gene expression; however, non-coding regulatory elements and epigenetic mechanisms, also represent a substantial proportion of GWAS hits reside in enhancers and regions of open chromatin that regulate expression through long-range interactions such as trans-QTLs not captured by standard cis-eQTL models, suggesting our models may incompletely instrument the full genetically regulated component of expression. Finally, cell-type heterogeneity in bulk tissue samples remains a known source of bias in eQTL mapping, and single-cell studies have confirmed that many disease-relevant eQTLs are detectable only in specific cell populations [104]. While we investigated protein–protein interaction networks and overrepresented pathways, other mechanisms such as non-coding regulatory elements, epigenetic modifications, and cell-type-specific expression patterns may also contribute to AD–cancer pleiotropy. Incorporating single-cell epigenomic and transcriptomic data into future analyses of this locus will therefore be essential to fully resolve the cell-type-specific regulatory mechanisms driving the observed pleiotropy. Lastly, while the in silico approaches used in our analysis reveal physiologically plausible overlaps, experimental validation in appropriate model systems is necessary to validate the functional relevance of the discovered candidate genes and pathways. For our prioritized candidate genes, the right validation methods could be (1) CRISPR-based gain- and loss-of-function screens in neurons and cancer cell lines made from iPSCs; (2) co-expression and pathway perturbation assays to see if the candidate genes interact with AD or oncogenic pathways; (3) conditional mouse models for brain-specific or tumor-relevant tissues; and (4) proteomics and transcriptomics profiling in post-mortem AD brain and matched tumor samples. For 19q13-specific candidates, luciferase reporter experiments to evaluate regulatory variant activity in pertinent cell types as used by Bekris et al. (2012) [105] would be very elucidative. In this study, we focused on late-onset AD and late-life cancers to minimize potential survivor bias. Particularly, PSEN1 and PSEN2 mutations, which drive autosomal-dominant early-onset AD, have been reported to be associated with various cancer types [106,107,108]; however, these were outside the scope of this investigation, as the available data remain limited [109].

## 4. Methods

### 4.1. Data Description

We focused primarily on age-associated cancer types, including breast cancer, prostate cancer, lung cancer, colorectal cancer, melanoma, basal cell carcinoma, bladder cancer, and endometrial cancer. For AD (N = 290,088), a meta-analysis was performed using METAL [110] to combine GWAS summary statistics from two databases: FinnGen [111] and Kunkle B. W. et al. [28]. Two well-powered AD GWAS datasets, FinnGen late-onset AD (with extended control exclusions) and the clinically diagnosed cohort reported by Kunkle B. W. et al. (2019) [28], showed a strong and statistically significant positive genetic correlation, rg = 0.880 (*p* = 7.59 × 10^−6^).

GWAS summary statistics for the cancer types were collected from multiple sources: breast cancer (N = 139,274) from Michailidou K. et al. [112], prostate cancer (N = 140,254) from Schumacher F. R. et al. [113], lung cancer (N = 85,716) from McKay J. D. et al. [19], colorectal cancer (N = 390,539) from FinnGen [111], melanoma (N = 434,871), basal cell carcinoma (N = 435,548), bladder cancer (N = 448,150) from Verma A. et al. [114], and endometrial cancer (N = 121,885) from O’Mara T. A. et al. [115]. All GWAS summary statistics utilized in this study were derived from individuals of European ancestry on hg19 build. A detailed summary of each GWAS dataset is provided in Supplementary Table S1.

### 4.2. SNP-Based Heritability Analysis (Observed Scale)

We estimated SNP-based heritability on the observed scale (h^2^_obs) for binary traits along with standard errors for AD and various cancer types using Linkage Disequilibrium Score Regression (LDSC) [116]. The observed-scale heritability (h^2^_obs) represents the proportion of phenotypic variance explained by common SNPs directly captured in the GWAS summary statistics. LDSC utilizes the relationship between LD scores and GWAS test statistics to partition heritability and account for potential confounding factors such as population stratification. For each phenotype, we used summary statistics from quality-controlled GWAS data. We employed pre-computed LD scores based on 1000 Genomes European reference populations to maintain consistency across analyses [117,118]. All analyses were performed using the LDSC software (v1.0.1) with default parameters as recommended by the developers (https://github.com/bulik/ldsc, accessed on 7 March 2026).

### 4.3. Global Genetic Correlation Analysis

To quantify the shared genetic architecture between AD and various cancer types, we performed global genetic correlation analyses using LDSC [116]. This method leverages the relationship between LD patterns and GWAS test statistics to estimate genetic correlations between traits. Prior to analysis, all datasets underwent standardized quality control on (GRCh37) genome build. We employed pre-computed European ancestry LD scores from the 1000 Genomes Project Phase 3 reference panel to ensure consistent LD estimates across all analyses. For each trait pair, we calculated the genetic correlation coefficient (rg) along with its standard error and corresponding *p*-value. (*p* < 0.05/n,). All analyses were conducted using LDSC software v1.0.1 (https://github.com/bulik/ldsc, accessed on 7 March 2026) with default parameters.

### 4.4. Local Genetic Correlation Analysis

To identify specific genomic regions exhibiting shared genetic architecture between AD and cancer types, we employed Local Analysis of co-Variance Association (LAVA), a method that detects and characterizes local genetic correlations between traits [53]. We analyzed AD GWAS against each of the eight cancer types. Prior to analysis, all summary statistics underwent standardized quality control procedures, including alignment to the GRCh37/hg19 reference genome, filtering for variants with minor allele frequency > 0.01, and removal of insertions/deletions and multi-allelic variants.

For the LAVA analysis, we utilized the 1000 Genomes Phase 3 European reference panel [117] to estimate the local LD structure across 2495 LD blocks. First, we conducted univariate testing to estimate heritability for each trait across all loci exhibiting nominal association (*p* < 0.05) for both phenotypes. In the second step, these loci underwent bivariate analysis to assess local genetic correlations. All LAVA analyses (v1.0.0; https://github.com/josefin-werme/LAVA, accessed on 8 March 2026) were performed using recommended default parameters. Visualization of local genetic correlation results was implemented using *ggraph* [119] in R to generate a network graph highlighting significant regions across the genome.

### 4.5. Transcriptome-Wide Association Study (TWAS)

We performed a TWAS using the FUSION method to find potential genes whose expression plays a mediating role in heritable phenotypic risk; this approach enhances interpretability compared to SNP-level GWAS [25,120]. This method leverages the cis-genetic aspect of gene expression, facilitating gene-level association testing while considering LD across SNPs. The FUSION method imputes tissue-specific gene expression levels using pre-computed gene expression prediction models trained on reference populations. Then, it tests for association with traits of interest.

To investigate the relationship between genetic variants and gene expression regulation, our approach used tissue-specific expression computational reference panels that had been previously developed to capture expression signatures across different tissues, derived from three major genomic consortia: the Genotype-Tissue Expression project version 8 (GTEx v8) [26], The Cancer Genome Atlas [121], and brain-specific expression information from dorsolateral prefrontal cortex from the CommonMind Consortium [122]. GTEx v8 tissues included were European ancestry samples for lung (N = 436), breast mammary (N = 329), prostate (N = 181), whole blood (N = 558), uterus (N = 107), adipose (subcutaneous and visceral) (N = 872), skin (sun-exposed lower leg and not sun-exposed suprapubic) (N = 938), and transformed fibroblasts (N = 403). For capturing gene regulation within tumor-adjacent and tumor tissues, TCGA expression reference weights were used. These datasets included lung adenocarcinoma (LUAD) (N = 500; features-2973), lung squamous cell carcinoma (LUSC) (N = 464; features-2544), breast invasive carcinoma (BRCA) (N = 1049; features-4464), prostate adenocarcinoma (PRAD) (N = 468; features-4675), skin cutaneous melanoma (SKCM) (N = 103; features-541), bladder urothelial carcinoma (BLCA) (N = 380; features-1677), and uterine corpus endometrial carcinoma (UCEC) (N = 171; features-518). To develop tumor-specific eQTL models, expression weights were calculated from tumor samples’ RNA-seq profiles and compared with germline genotyping data [121,123]. By using TCGA-based expression panels, TWAS analysis may more easily probe cancer-specific regulatory mechanisms. To facilitate brain-specific gene expression modeling, which is especially important for neurodegenerative disease phenotypes like AD, DLPFC (Dorsolateral Prefrontal Cortex) tissue weights from the CommonMind Consortium [122] were used. Expression weights for these genes were downloaded from the publicly available FUSION website (http://gusevlab.org/projects/fusion/, accessed on 8 March 2026), which hosts precomputed weights for GTEx, CMC, and TCGA datasets.

We conducted the TWAS using the described precomputed expression weights, LD reference panels from 1000 Genomes Project Phase 3 European ancestry samples [117], and GWAS of AD and cancer. TWAS associations were tested for thousands of genes across several healthy and cancer tissues. We considered genes with nominally significant associations (*p* < 0.05) for downstream analyses and inverse Z scores between the overlapping genes.

### 4.6. Protein–Protein Interaction Network Construction and Pathway Analysis

We performed a pathway enrichment analysis on the nominally significant genes (*p* < 0.05) from the TWAS to find the biological pathways and molecular processes. We leveraged the IMEx database to create protein–protein interaction (PPI) networks [52], implemented within the *networkcatalyst* package R 4.4 [124]. The network was reduced to minimum network, which retains prioritizes proteins that connect at least two or more genes from the query genes. We did pathway enrichment analysis on the largest protein cluster using Gene Ontology (GO):Biological Process (GO:BP), and corrected for multiple testing using FDR *p* > 0.05.

## 5. Conclusions

To summarize, we identified that the *APOE* locus (19q13.31–19q13.32) shares inverse genetic effects between several cancer types and AD. Further TWAS analysis demonstrated that the genes in the *APOE* regions are also crucial for mediating pleiotropic effects whose genetically regulated expression patterns overlapped between AD and cancers. We also highlight several other genes involved in crucial roles of cell-cycle and apoptotic regulation, which are likely to differentiate between AD and cancer pathology. The possibility of antagonistic pleiotropy, which holds that depending on the tissue and cellular context, shared genetic variants may have opposite effects on cancer risk and neurodegeneration, is supported by these data. Our research gives new insights into the molecular connections between AD and cancer, but further functional validation is needed. It also presents opportunities for finding potential therapeutic targets that are relevant to both neurodegeneration and oncology.

## Data Availability

The original contributions presented in this study are included in the article/Supplementary Materials. Further inquiries can be directed to the corresponding authors.

## Supplementary Materials

The following supporting information can be downloaded at: https://www.mdpi.com/article/10.3390/ijms27062900/s1.
