# Supplementary material for: Locus- and Gene-Level Insights into the Inverse Association Between Alzheimer’s Disease and Cancer
Source: Int J Mol Sci. 2026 Mar 23;27(6):2900. doi: 10.3390/ijms27062900 (PMC13026669; doi:10.3390/ijms27062900)
Supplement: Supplementary file 1 [file ijms-27-02900-s001.zip › Supplementary Fig 1 & 2 with captions.pdf]

Supplementary Figure S1 Nominally significant shared genes between Alzheimer's disease and cancers identified by TWAS across multiple tissues.

a.

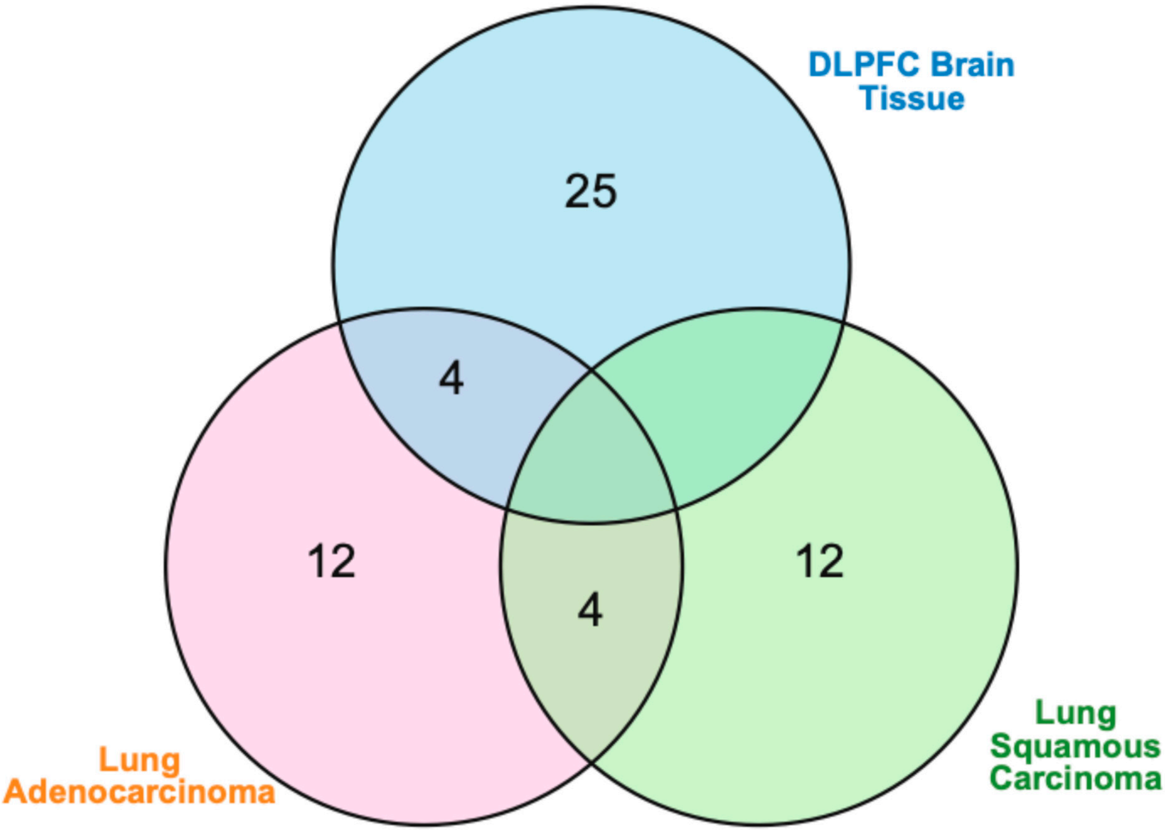

a. Shared genes between Alzheimer's disease and lung cancer identified by TWAS across multiple tissues.

The Venn diagram shows the overlap of AD–lung cancer associated genes observed in three tissue types: dorsolateral prefrontal cortex (DLPFC, blue), lung adenocarcinoma (pink), and lung squamous carcinoma (green). The equal overlap occurs between DLPFC and lung adenocarcinoma (4 genes) as well as lung adenocarcinoma, and lung squamous carcinoma (4 genes). Created in BioRender. Debnath, D. (2026) https://BioRender.com/fzi33lh, accessed on 18 March 2026.

b.

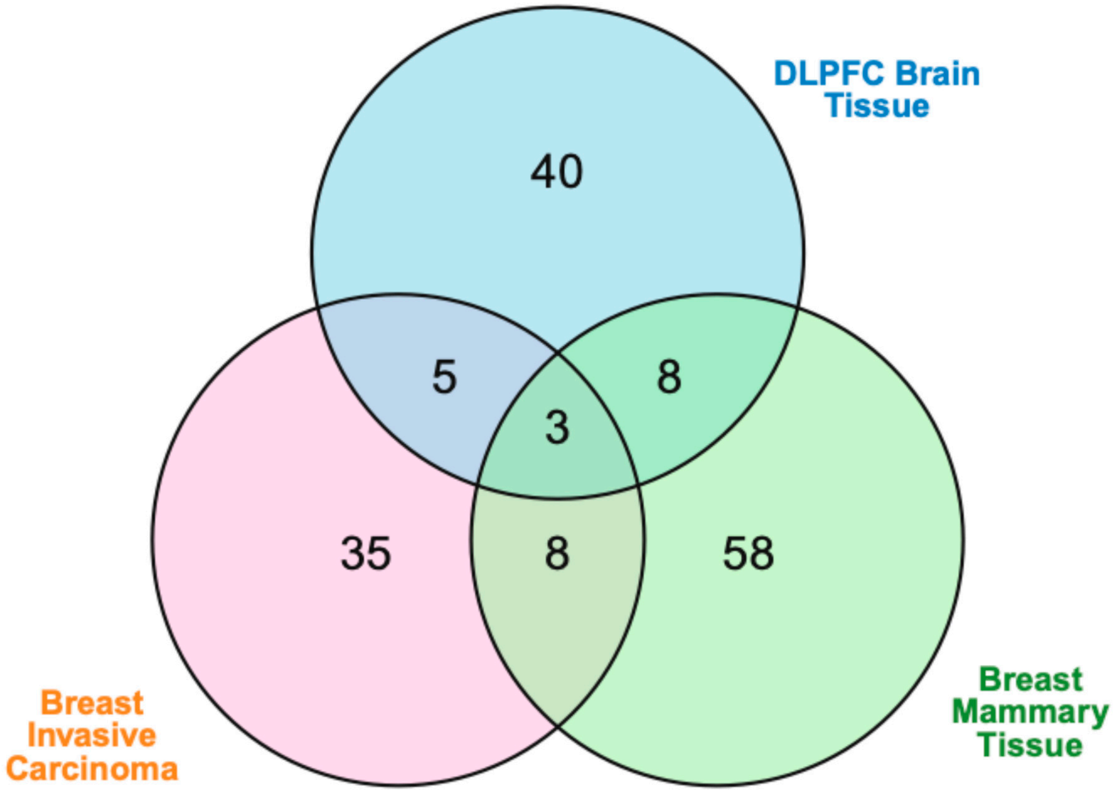

b. Shared genes between Alzheimer's disease and breast cancer identified by TWAS across multiple tissues.

The Venn diagram shows the overlap of AD–breast cancer associated genes observed in three tissue types: dorsolateral prefrontal cortex (DLPFC, blue), breast invasive carcinoma (pink), and breast mammary tissue (green). The equal overlap occurs between DLPFC and breast mammary tissue (8 genes) as well as breast invasive carcinoma, and breast mammary tissue (8 genes). Five genes shared between DLPFC and breast invasive carcinoma tissue, and three genes shared across all tissues investigated. Created in BioRender. Debnath, D. (2026) https://BioRender.com/678ynb8, accessed on 18 March 2026.

c.

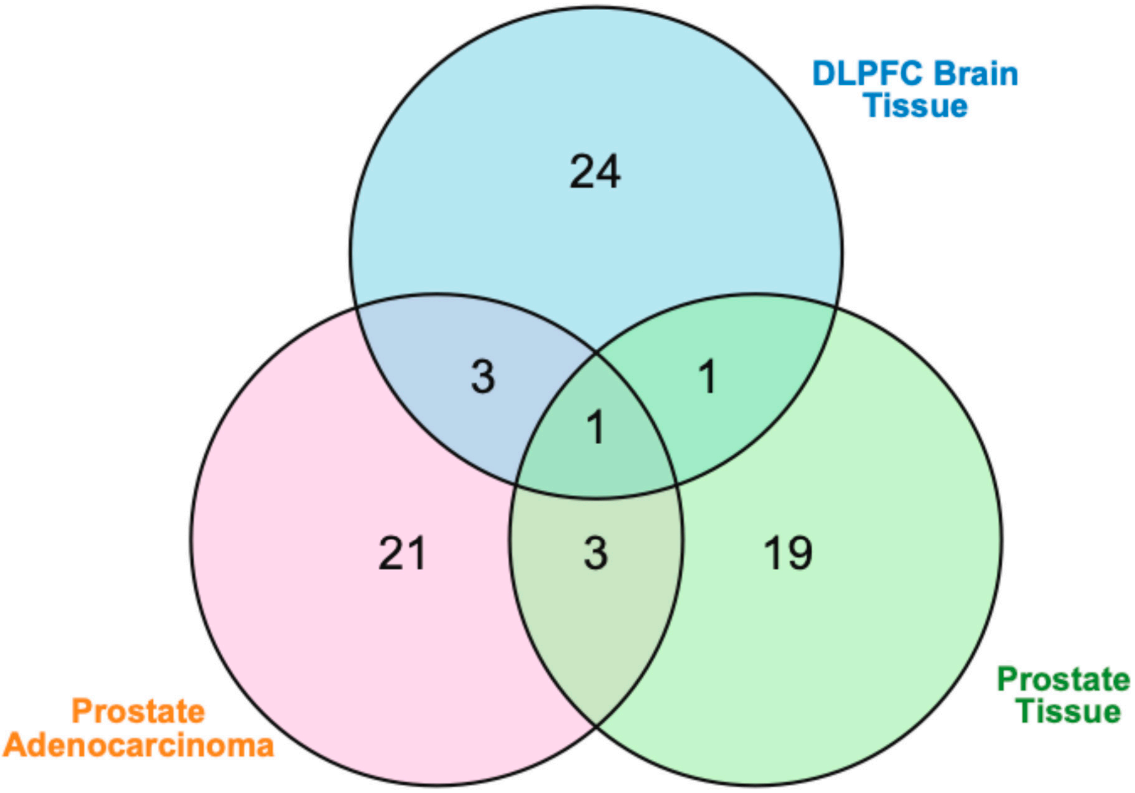

c. Shared genes between Alzheimer's disease and prostate cancer identified by TWAS across multiple tissues.

The Venn diagram shows the overlap of AD–prostate cancer associated genes observed in three tissue types: dorsolateral prefrontal cortex (DLPFC, blue), prostate adenocarcinoma (pink), and prostate tissue (green). The equal overlap occurs between DLPFC and prostate adenocarcinoma (3 genes) as well as prostate adenocarcinoma, and prostate tissue (3 genes). One gene shared between DLPFC and prostate tissue, as well as across all tissues investigated. Created in BioRender. Debnath, D. (2026) https://BioRender.com/xh21egw, accessed on 18 March 2026.

d.

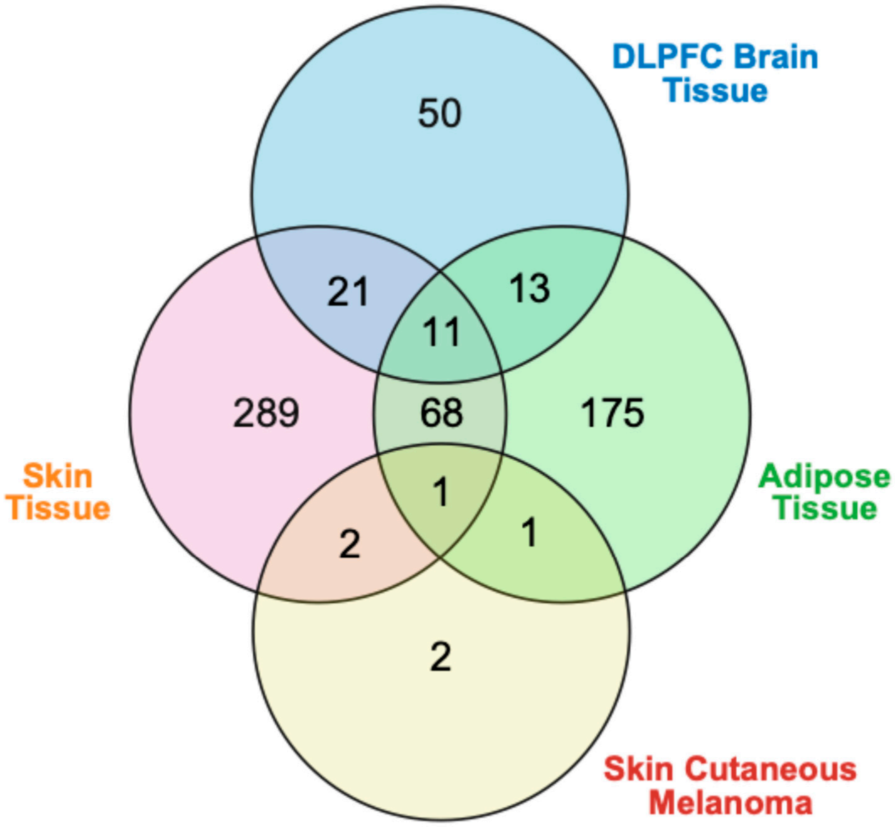

d. **Shared genes between Alzheimer's disease and melanoma identified by TWAS across multiple tissues.**

The Venn diagram shows the overlap of AD–melanoma associated genes observed in four tissue types: dorsolateral prefrontal cortex (DLPFC, blue), adipose tissue (green), skin tissue (pink), and skin cutaneous melanoma (yellow). The largest overlap occurs between skin and adipose tissue (68 genes), while 11 genes are shared between DLPFC and both skin and adipose tissue. 21 genes shared between DLPFC & skin tissue; 13 genes shared between DLPFC & adipose tissue; 2 genes shared between skin tissue & skin cutaneous melanoma; only 1 gene shared between adipose, skin and skin cutaneous melanoma. Created in BioRender. Debnath, D. (2026) https://BioRender.com/z8d0mif, accessed on 18 March 2026.

e.

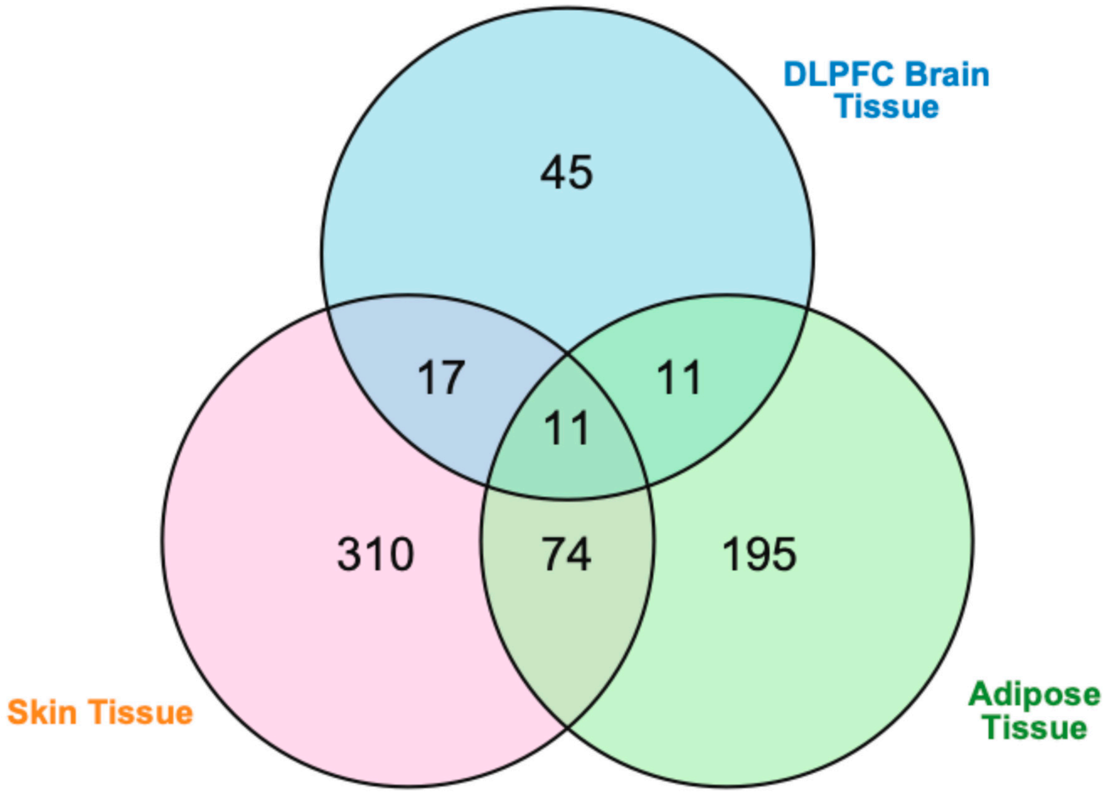

e. Shared genes between Alzheimer's disease and basal cell carcinoma identified by TWAS across multiple tissues.

The Venn diagram shows the overlap of AD–basal cell carcinoma associated genes observed in three tissue types: dorsolateral prefrontal cortex (DLPFC, blue), skin tissue (pink), and adipose tissue (green). 17 genes overlapped between DLPFC & skin tissue; 74 genes between skin & adipose tissues; equal number of genes (11 genes) shared between DLPFC & adipose tissue as well as shared across all tissues tested. Created in BioRender. Debnath, D. (2026) https://BioRender.com/g49ltw2, accessed on 18 March 2026.

f.

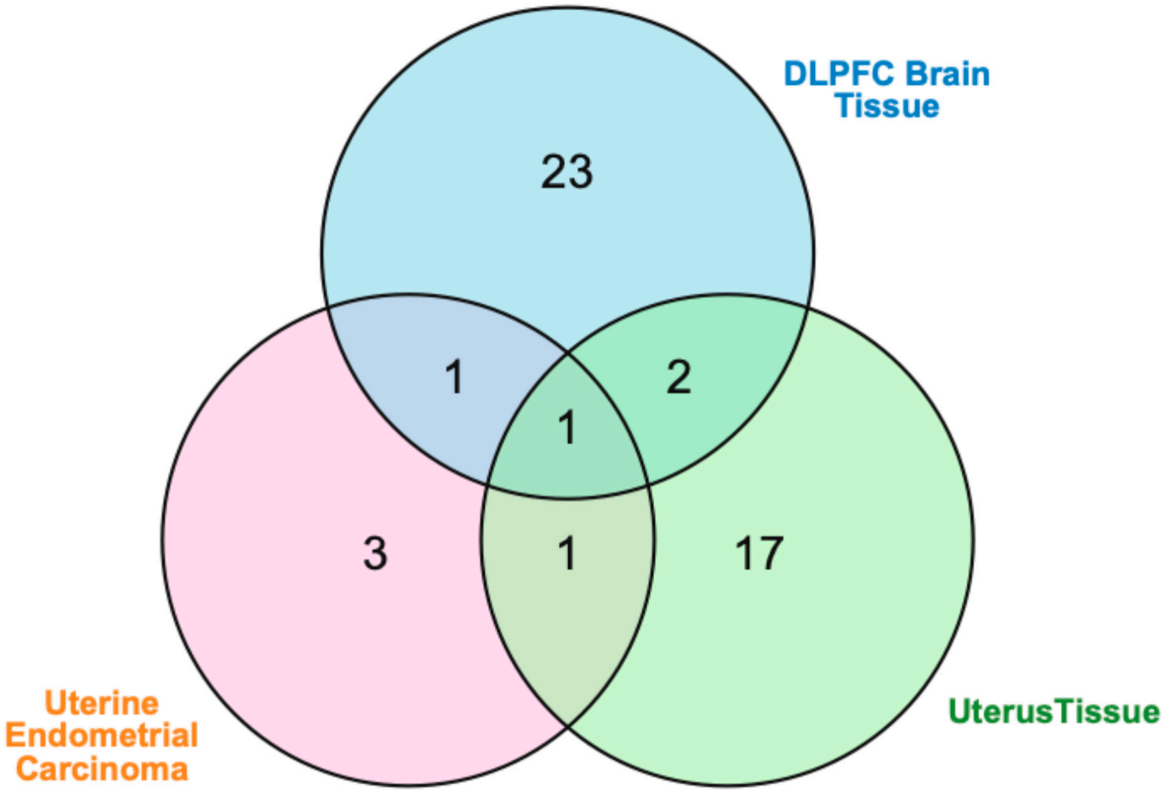

f. Shared genes between Alzheimer's disease and endometrial cancer identified by TWAS across multiple tissues.

The Venn diagram shows the overlap of AD–endometrial cancer associated genes observed in three tissue types: dorsolateral prefrontal cortex (DLPFC, blue), uterine endometrial carcinoma (pink), and uterus tissue (green). Equal number of genes (only 1 gene) shared between DLPFC & uterine endometrial carcinoma; uterine endometrial carcinoma & uterus tissue as well as shared across all tissues tested. 2 genes shared between DLPFC & uterus tissue. Created in BioRender. Debnath, D. (2026) https://BioRender.com/swllpry, accessed on 18 March 2026.

g.

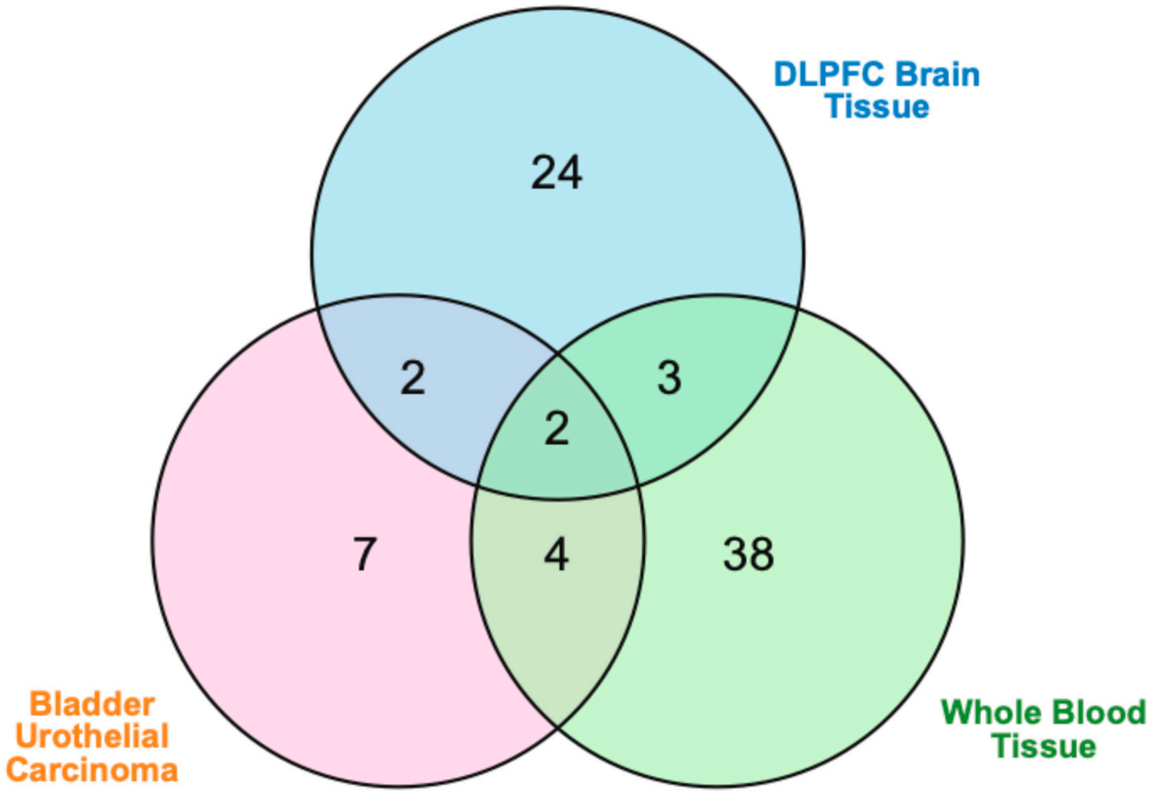

g. Shared genes between Alzheimer's disease and bladder cancer identified by TWAS across multiple tissues.

The Venn diagram shows the overlap of AD–bladder cancer associated genes observed in three tissue types: dorsolateral prefrontal cortex (DLPFC, blue), bladder urothelial carcinoma (pink), and whole blood tissue (green). Equal number of genes (2 genes) shared between DLPFC & bladder urothelial carcinoma; as well as shared across all tissues tested. 3 genes shared between DLPFC & whole blood tissue; 4 genes shared between bladder urothelial carcinoma & whole blood tissue. Created in BioRender. Debnath, D. (2026) https://BioRender.com/sqosmfp, accessed on 18 March 2026.

Supplementary Figure S2 Overlap between LAVA local genetic correlation regions and TWAS gene-level associations for Alzheimer's disease and cancers.

a.

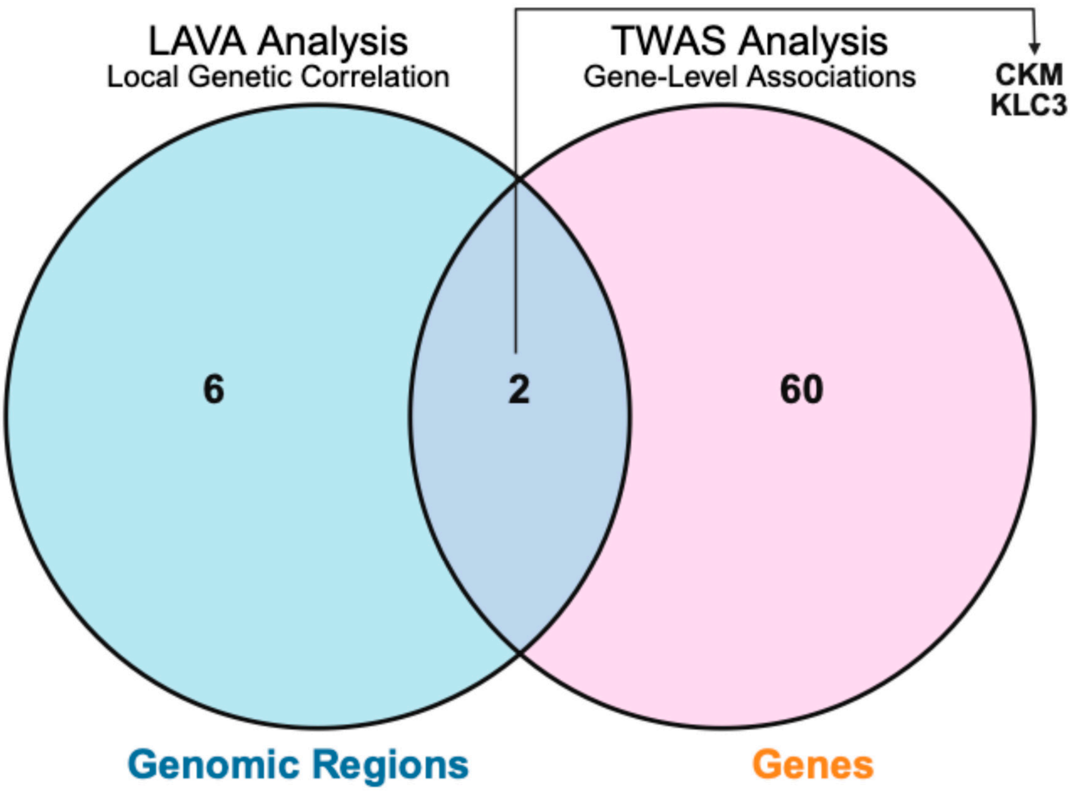

a. Overlap between TWAS gene-level associations and LAVA local genetic correlation regions for Alzheimer's disease and lung cancer.

The Venn diagram shows the intersection of genomic findings from two complementary analytical approaches. The left circle (blue) represents genomic regions identified through LAVA showing significant local genetic correlation between Alzheimer's disease and lung cancer (6 regions). The right circle (pink) represents genes identified through TWAS showing significant gene-level associations with both traits (60 genes). The intersection shows 2 genes that fall within genomic regions on 19q13.32 exhibiting significant local genetic correlation, including *CKM* and *KLC3*. Created in BioRender. Debnath, D. (2026) https://BioRender.com/xzmy30k, accessed on 18 March 2026.

b.

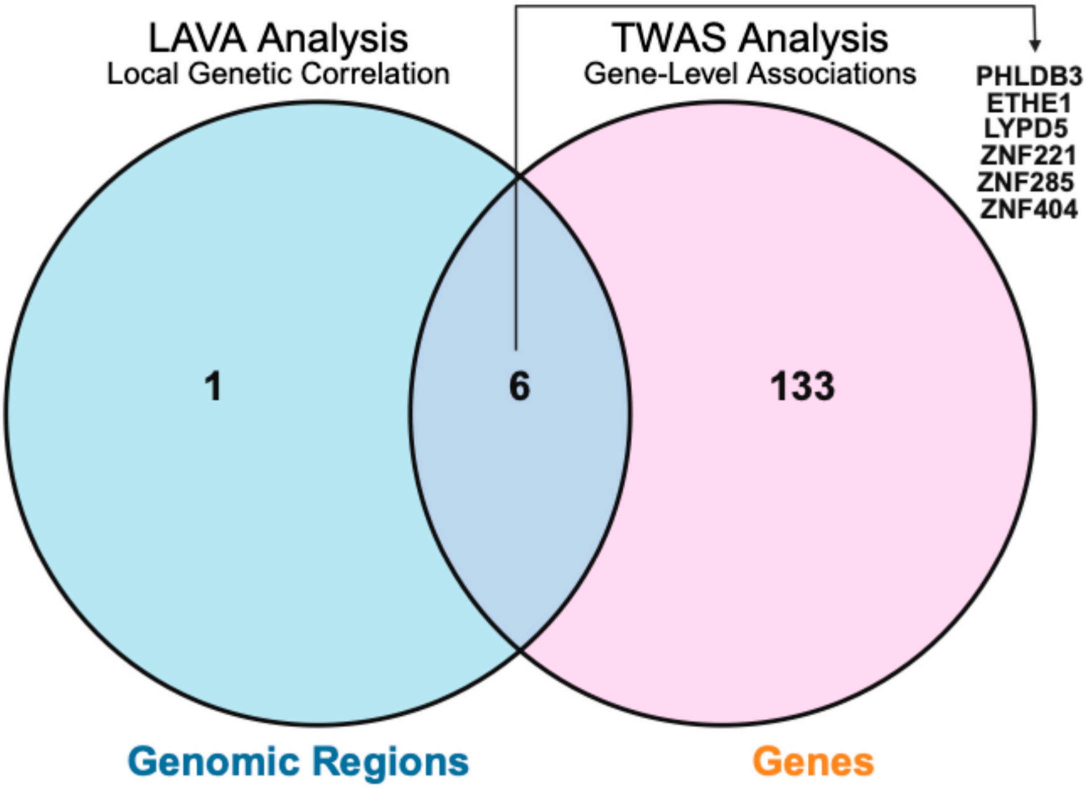

b. Overlap between TWAS gene-level associations and LAVA local genetic correlation regions for Alzheimer's disease and breast cancer.

The Venn diagram shows the intersection of genomic findings from two complementary analytical approaches. The left circle (blue) represents genomic regions identified through LAVA showing significant local genetic correlation between Alzheimer's disease and breast cancer (1 region). The right circle (pink) represents genes identified through TWAS showing significant gene-level associations with both traits (133 genes). The intersection shows 6 genes that fall within genomic regions on 19q13.31 exhibiting significant local genetic correlation, including *PHLDB3*, *ETHE1*, *LYPD5*, *ZNF221*, *ZNF285* and *ZNF404*. Created in BioRender. Debnath, D. (2026) https://BioRender.com/yjdgcgm, accessed on 18 March 2026.

c.

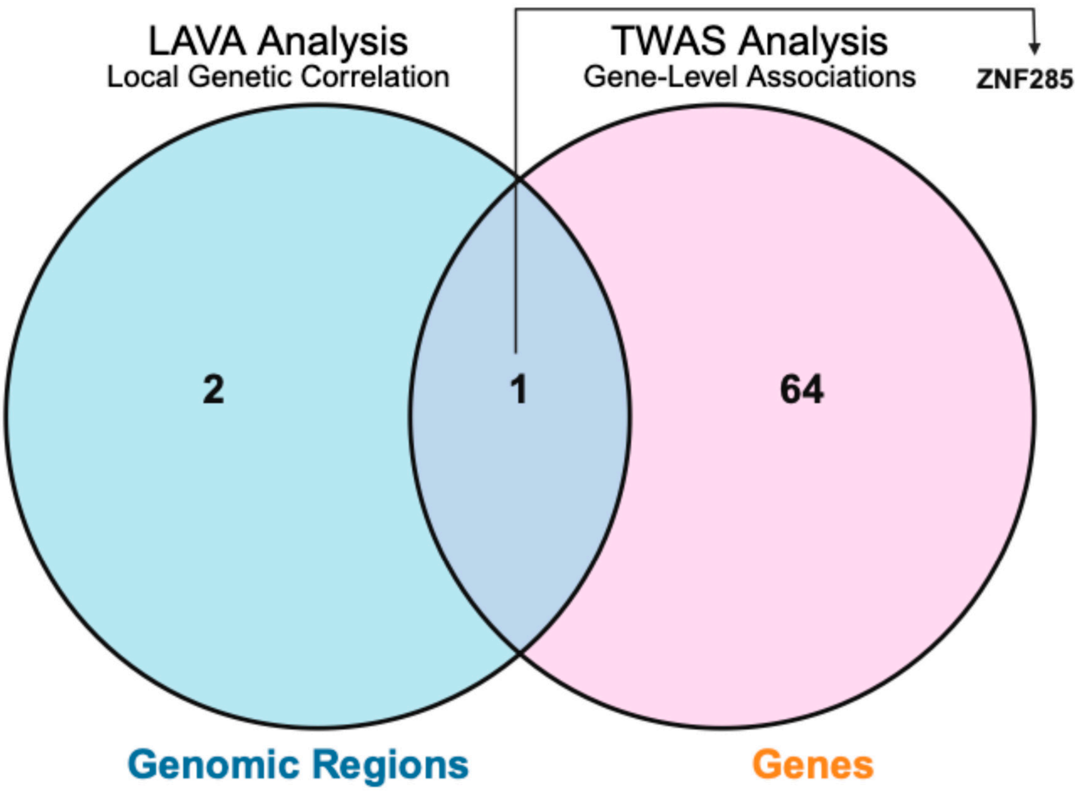

c. Overlap between TWAS gene-level associations and LAVA local genetic correlation regions for Alzheimer's disease and prostate cancer.

The Venn diagram shows the intersection of genomic findings from two complementary analytical approaches. The left circle (blue) represents genomic regions identified through LAVA showing significant local genetic correlation between Alzheimer's disease and prostate cancer (2 regions). The right circle (pink) represents genes identified through TWAS showing significant gene-level associations with both traits (64 genes). The intersection shows 1 gene that fall within genomic regions on 19q13.31 exhibiting significant local genetic correlation, which include *ZNF285*. Created in BioRender. Debnath, D. (2026) https://BioRender.com/khzco5x, accessed on 18 March 2026.

d.

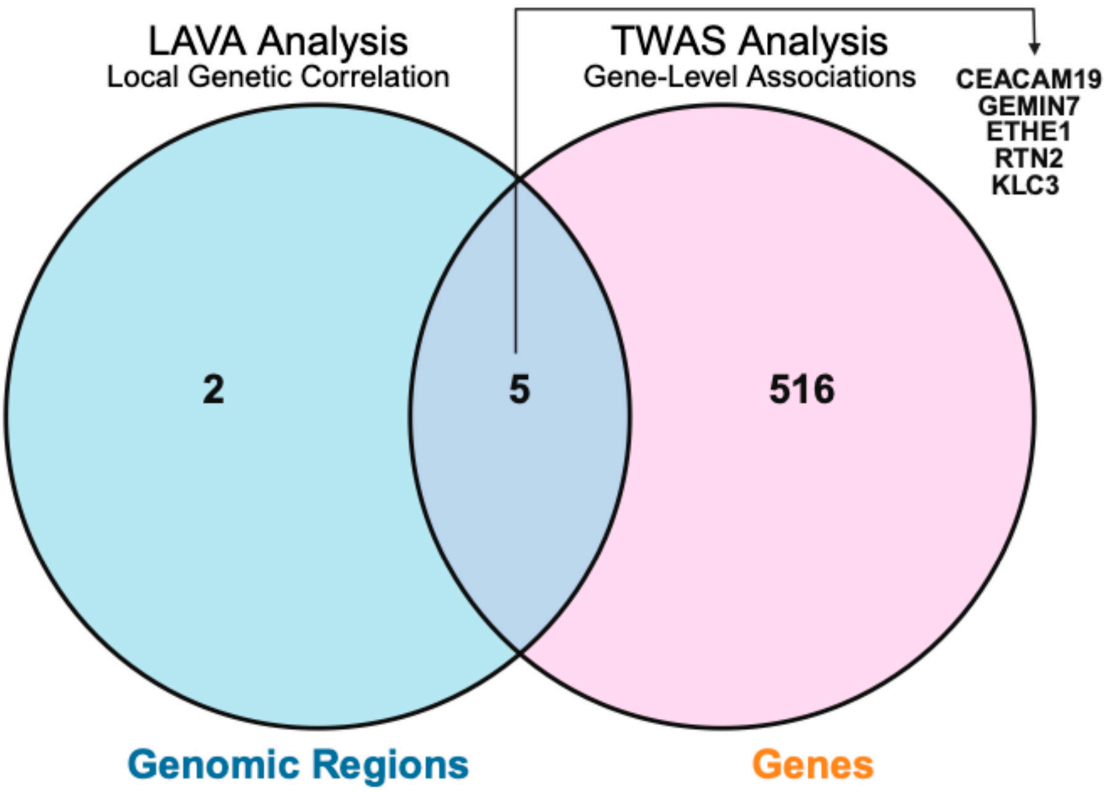

d. Overlap between TWAS gene-level associations and LAVA local genetic correlation regions for Alzheimer's disease and melanoma.

The Venn diagram shows the intersection of genomic findings from two complementary analytical approaches. The left circle (blue) represents genomic regions identified through LAVA showing significant local genetic correlation between Alzheimer's disease and melanoma (2 regions). The right circle (pink) represents genes identified through TWAS showing significant gene-level associations with both traits (516 genes). The intersection shows 5 gene that fall within genomic regions on 19q13.31-19q13.32 exhibiting significant local genetic correlation, including *CEACAM19*, *GEMIN7*, *ETHE1*, *RTN2*, and *KLC3*. Created in BioRender. Debnath, D. (2026) https://BioRender.com/vjw6wr7, accessed on 18 March 2026.
